# One-step antimicrobial peptide immobilization technique: universal compatibility with clinical bone cements

**DOI:** 10.1039/d6ra08377c

**Published:** 2026-11-12

**Authors:** Chen Wang, Hao Lin, Bo Lu, Xin Wang, Yiming Li, Zhaoyang Li, Jianjun Chu, Yang Xu, Fang He

**Affiliations:** a Hefei BOE Hospital, Shanghai University Medical College Hefei China xuyang@hfut.edu.cn; b The Second People's Hospital of Hefei, Anhui Medical University Hefei China chujianj@mail.ustc.edu.cn; c School of Food and Biological Engineering, Hefei University of Technology Hefei China hefang8@126.com; d School of Materials Science and Engineering, Tianjin University Tianjin China

## Abstract

Nisin-based antimicrobial peptide (AMP) immobilization represents a promising strategy for antibacterial bone cements. This one-step approach fits standard surgical workflows and addresses drawbacks of antibiotic-loaded cements, such as burst drug release, short-term antibacterial activity and compromised mechanical properties. Nevertheless, its compatibility with clinical cements for translation has not been verified. Here, we covalently anchored nisin onto three clinical cements during *in situ* polymerization and assessed pre-clinical performances. The modified cements maintained ISO-compliant compressive strength and MMA release profiles remained largely unaltered. They yielded 94–100% contact-killing efficacy against *Staphylococcus aureus* without peptide leaching. Moreover, the treated cements maintained desirable handling properties and biocompatibility. This work validates the broad compatibility of this one-step technique. Featuring low-cost nisin, it offers an accessible translational anti-infective cement strategy, especially for resource-limited regions.

## Abstract 2

This study validates one-step antimicrobial peptide nisin-based modification compatible with clinical cements. Modified cements retain suitable handling and biocompatibility. This low-cost strategy facilitates anti-infective bone cement translation.

## Graphical abstract

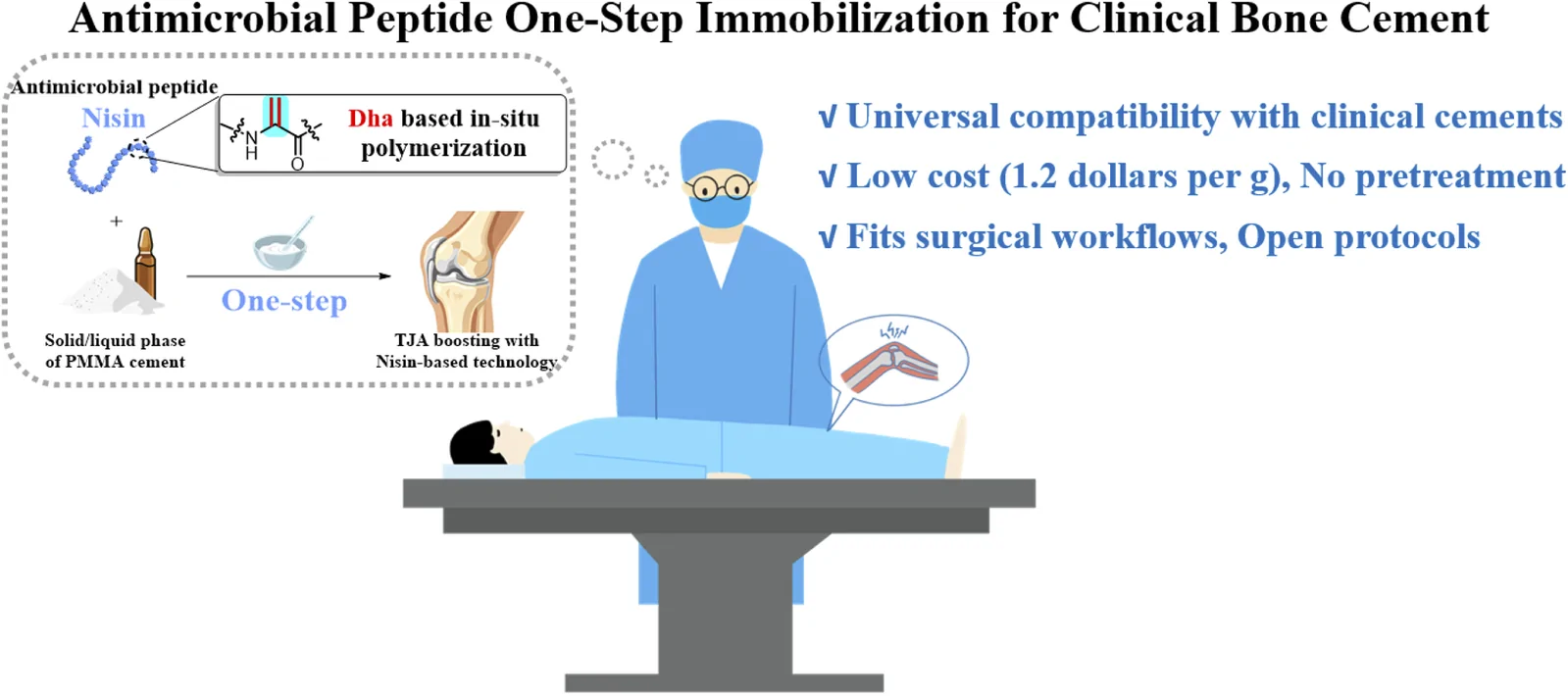

## 1 Introduction

Driven by global aging, war trauma and other factors, the volume of orthopedic procedures has risen continuously.^1–4^ In China, total hip and knee arthroplasties exceeded 950 000 cases in 2019; in the United States, annual joint arthroplasty volume is projected to reach 4 million cases by 2030.^5,6^ However, despite preoperative prophylactic antibiotic therapy, implant-associated infections remain highly prevalent, with rates exceeding 25% in complex open fractures or revision surgeries.^7^ Among these, periprosthetic joint infection (PJI) is the most devastating post-TJA adverse event: it is a leading cause of revision arthroplasty, with a reported recurrence rate of 16% and all-cause mortality up to 2.5%.^8–10^ To address these unmet clinical challenges, antibiotic-loaded polymethylmethacrylate (PMMA) bone cement (ALBC) has emerged as the cornerstone and clinical gold standard for preventing and managing orthopedic implant-associated and post-traumatic infections.^11–18^ However, it is plagued by inherent initial burst release, a critical flaw that causes four major drawbacks: an effective antibacterial time window as short as one week, intrinsic biotoxicity, induction of antibiotic resistance, and compromised mechanical strength.^19–23^

Recently, antibacterial agent immobilization technology has expanded the research horizons for rational design of high-performance bone cements.^23–36^ Covalently tethering antibacterial agents to the cement matrix delivers two pivotal advancements. These include complete circumvention of inherent burst release and avoidance of cement mechanical deterioration. The pioneering work of Sydlik *et al.* established the robust proof-of-concept for this strategy, fabricating modified bone cements with immobilized small-molecule drugs and gentamicin that showed remarkable superiority over conventional ALBC, significantly advancing the field.^23–25^ Building on this foundational progress, we sought to address the remaining bottlenecks hindering clinical translation. Specifically, multi-step chemical modification of existing immobilization systems poses notable challenges to intraoperative point-of-care implementation, especially in resource-limited settings without access to specialized equipment and expertise.

In 2025, our group developed a one-step fabrication technique for antibacterial bone cement based on nisin, a natural antimicrobial peptide (AMP) and US Food and Drug Administration (FDA)-approved food additive with a long history of global safe use (Fig. 1).^26^ Nisin possesses a unique molecular structure containing two free dehydroalanine (Dha) residues, each carrying a highly reactive α, β-unsaturated double bond.^37–44^ This feature enables nisin to act as a comonomer and copolymerize into the cement matrix during *in situ* polymerization, thereby achieving one-step covalent immobilization without additional chemical modification. This breakthrough technology enables a barrier-free fabrication route for high-performance antibacterial bone cement. It adopts low-cost commercial raw materials and is fully compatible with routine surgical procedures, requiring no specialized skills or equipment for clinical staff. Meanwhile, the excellent safety profile of nisin further strengthens its potential for future clinical translation.

**Fig. 1:**
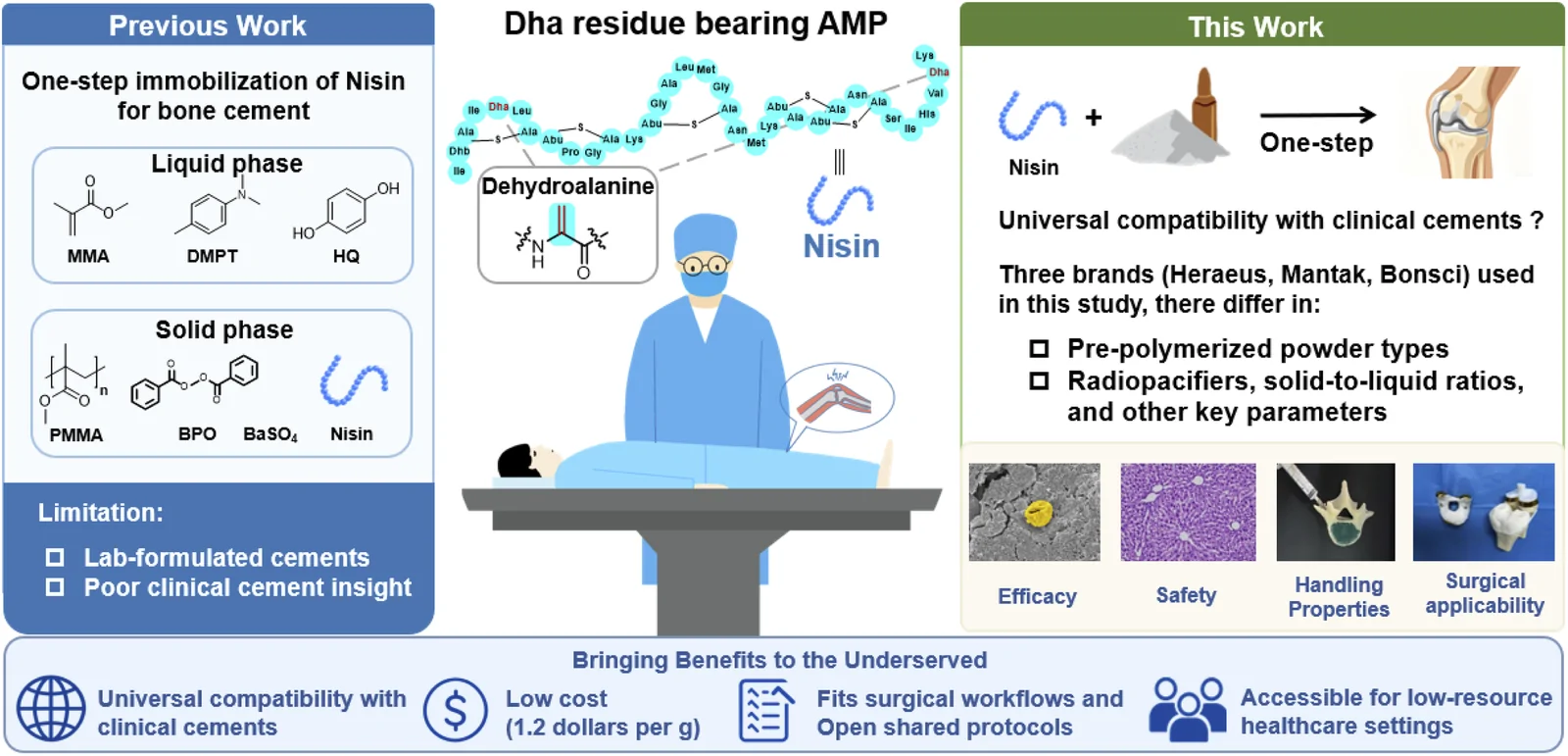
Overview of previous one-step immobilization of nisin and the present work for clinical bone cement compatibility evaluation.

However, a critical question of whether this nisin-based AMP-immobilized fabrication technique is compatible with commercially available clinical bone cements remains unresolved. This directly determines the clinical translational potential of this nisin-based technique and its capacity to benefit a broader patient population, particularly in low- and middle-income countries (LMICs) and regions affected by prolonged armed conflict. We therefore aim to validate the universal applicability of this easy-to-implement technique, and provide robust preclinical evidence to comprehensively verify its efficacy and biosafety (Fig. 1). We are willing to share this protocol free of charge with frontline clinicians and researchers in resource-limited settings, with the ultimate goal of benefiting more patients globally.

## 2 Materials and methods

### 2.1 Materials

In this study, three clinically applied PMMA bone cements were selected, namely PALACOS® MV manufactured by Heraeus Medical GmbH (Germany), MT-HA produced by Shandong Mingde Biomedical Engineering Co., Ltd (China), and J-MV-40 supplied by Beijing Bangsai Technology Co., Ltd nisin, α-tricalcium phosphate (α-TCP), and Gentamicin sulfate (GS) were purchased from Macklin Biochemical Co., Ltd MC3T3-E1 cells and their special complete culture medium were obtained from Procell Life Science & Technology Co., Ltd (Wuhan). *Staphylococcus aureus* (*S. aureus*, ATCC 25923) was obtained from the Department of Clinical Laboratory of the Second People's Hospital of Hefei. Specific pathogen-free (SPF) C57BL/6 laboratory mice were supplied by the Laboratory Animal Center of Anhui Medical University. The MTS809 material testing machine used in the experiment was provided by the University of Science and Technology of China, and the gas chromatograph (GC) was offered by Hefei University of Technology. Conventional laboratory instruments including centrifuges, CO_2_ incubators, clean benches and microplate readers were mainly applied for cell culture, bacterial culture, as well as related physicochemical and biological detection experiments.

### 2.2 Bone cement preparation

PMMA bone cement and calcium phosphate cement (CPC) cement were prepared according to the formulas listed in Tables S1 and S2, respectively. To unify experimental conditions, identical solid and liquid dosages were adopted for all cements, with 638 mg solid powder mixed with 385 µL liquid component in each group. The unified ratio does not replace the official proportion recommended by manufacturers, nor does this study aim to evaluate the optimal performance of each cement under factory-specified ratios. This fixed solid–liquid ratio helps establish a standardized, convenient and reproducible experimental system. On this basis, the effects of nisin incorporation on the physicochemical properties, handling characteristics and biological performance of different clinical PMMA bone cements were preliminarily investigated.

According to the formulation, nisin was weighed and mixed with the powder component of PMMA cement to ensure its uniform dispersion in the powder phase. Afterwards, 385 µL liquid component was added, and the mixture was stirred at a constant speed for 30 seconds. When the cement reached the dough stage, the obtained pasty mixture was poured into customized molds for shaping. In this study, the nisin-modified cements were designated as PALACOS® MV-N, MT-HA-N, and J-MV-40-N, while the corresponding cements without nisin modification were designated as PALACOS® MV, MT-HA, and J-MV-40.

### 2.3 Measurement of mechanical properties

PMMA bone cement was prepared according to the method described in Section 2.2, and compressive tests were conducted in accordance with ISO 5833 to evaluate the effect of nisin incorporation on the mechanical properties of the cement. Five cylindrical specimens were prepared for each group. Prior to testing, all specimens were polished with 1000-grit sandpaper to ensure flat and parallel upper and lower surfaces and reduce experimental errors. The compressive mechanical tests were performed using an MTS809 microcomputer-controlled material testing machine (Bose Corporation, USA) at a loading rate of 20 mm min^−1^. The stress–strain curves of specimens in each group were synchronously collected and recorded during the tests, and the compressive strength of bone cement in each group was subsequently calculated based on the obtained curves.

### 2.4 Methyl methacrylate (MMA) content determination

To explore the effect of nisin addition on the release behavior of MMA monomers from PMMA cement, GC was used to determine the release of free MMA during the immersion process, with three specimens prepared for each group. Prior to the experiment, 5 mL of deionized water was added into 10 mL glass test tubes and preheated in an oven at 37 °C for 30 min. After solid–liquid mixing of bone cement components, the mixtures were cured at room temperature for 30 min, and the mass of each specimen was accurately weighed and recorded. Subsequently, the specimens were immersed in preheated deionized water at a constant temperature of 37 °C, and the immersion time was recorded immediately after placement. The extracts were collected at immersion durations of 1 h, 24 h and 72 h. At each preset time point, the bone cement specimens were taken out and all extracts were collected. Without rinsing, the specimens were directly transferred into new glass test tubes containing 5 mL preheated deionized water for continuous constant-temperature immersion until the next sampling time point. The collected extracts were sealed and preserved, and ethyl acetate was adopted to extract MMA monomers in the soaking solution. After extraction, the samples were detected and analyzed by gas chromatography. An HP-5 capillary column was used with an injection volume of 2 µL. The programmed temperature procedure was set as follows: the initial temperature was 45 °C and maintained for 2.75 min, then increased to 250 °C at a heating rate of 5 °C min^−1^, and kept at 250 °C for 4 min. In addition, the standard curve of MMA was established on the same day for the quantitative analysis of free MMA content.

Gradient standard solutions of MMA with concentrations of 10, 30, 50, 100, 300, 500 and 1000 µg mL^−1^ were prepared and determined under identical GC conditions. The standard curve was established *via* linear fitting between MMA concentration and corresponding peak area for the quantitative analysis of free MMA. The single release amount of MMA was expressed as the mass of released MMA per unit mass of bone cement at each time interval, with the unit of mg g^−1^. The calculation formula is as follows:
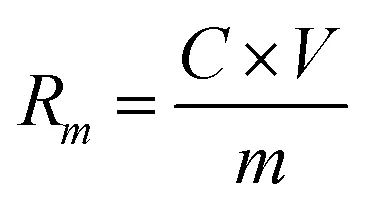
*R*_*m*_ is the cumulative release amount of MMA within the corresponding period (mg g^−1^); *C* is the MMA concentration in the soaking solution calculated from the standard curve (mg mL^−1^); *V* stands for the volume of collected soaking solution, which is 5 mL in this experiment; *m* is the mass of bone cement sample (g).

### 2.5 Scanning electron microscopy (SEM)

Two square specimens (20 mm × 20 mm × 2 mm) were prepared for each of the PMMA, GS-loaded, and nisin-modified bone cements. One specimen was fractured in liquid nitrogen and immersed in normal saline at room temperature for 7 days, while the other remained unsoaked. After soaking, all specimens were sputter-coated with gold and observed by SEM (ZEISS Sigma 360, Germany) to characterize the cross-sectional morphology of the bone cement. Additional specimens (5 mm × 5 mm × 2 mm) were prepared. After immersion in normal saline for 1 d and UV sterilization, the specimens were incubated in *S. aureus* suspension at 37 °C with 5% CO_2_ for 6 h. Subsequently, the specimens were rinsed to remove non-adherent bacteria and fixed overnight in 2.5% glutaraldehyde at 4 °C. After gold sputtering, the morphology and distribution of bacteria adhered to the cement surface were observed by SEM.

### 2.6 Antibacterial activity

*S. aureus* was selected as the test strain. Three types of nisin-modified PMMA bone cement and the corresponding bare cement were prepared for inhibition zone and surface antibacterial tests, while additional CPC cement and nisin-containing CPC cement (CPC-N) were included in the inhibition zone test. Disc-shaped samples (6 mm × 3 mm) were used for the inhibition zone test, while cylindrical samples (6 mm × 12 mm) were used for the surface antibacterial assay. *S. aureus* was resuscitated and subcultured three consecutive times to prepare a bacterial suspension with a concentration of 5 × 10^7^ CFU mL^−1^. For the inhibition zone test, 100 µL of bacterial suspension was evenly spread onto the surface of nutrient agar plates. Subsequently, UV-sterilized samples were placed in the center of the plates and incubated at 37 °C with 5% CO_2_ for 24 h. The bacterial growth around the samples and the formation of inhibition zones were observed and recorded. For the surface antibacterial assay, sterilized samples were immersed in 1 mL of bacterial suspension and incubated at 37 °C with 5% CO_2_ for 6 h. After incubation, the cement specimens were transferred to sterile centrifuge tubes and gently rinsed three times with deionized water to remove planktonic bacteria. Afterwards, the specimens were placed in centrifuge tubes containing 5 mL of normal saline and subjected to ultrasonic treatment for 3 min to detach the adherent bacteria from the material surface. A total of 40 µL eluate was diluted with 4 mL of normal saline. After thorough mixing, 40 µL of the diluted solution was evenly spread onto nutrient agar plates, followed by incubation at 37 °C with 5% CO_2_ for 24 h. Finally, bacterial colonies on the plates were observed and colonies were counted, and the antibacterial rate of each group was calculated based on the counting results.

### 2.7 Determination of injectability

To mimic the porous architecture of cancellous bone, porous foam materials were implanted into the interior of the 3D-printed vertebral model. Subsequently, the prepared MT-HA-N bone cement was injected into the model. After complete curing of the bone cement, the excess foam material on the model surface was removed, and the filling morphology and distribution of the bone cement within the porous structure were observed. In addition, fresh porcine vertebrae were used to further evaluate the filling performance of the bone cement in native bone tissue. The muscle and soft tissues attached to the vertebral surface were first carefully removed. Cylindrical working channels were then established along the pedicle direction using a surgical drill. The channels were approximately 4 mm in diameter and 50 mm in depth. Subsequently, the prepared MT-HA-N bone cement was injected into the channels. After complete curing, X-ray imaging was performed to observe the filling behavior and distribution of the bone cement within the vertebral channels.

### 2.8 Handling properties and joint prosthesis fixation model test

All experiments were performed at a temperature of 21 ± 1 °C and relative humidity of 40% ± 5%. 1 g of bone cement was weighed, and timing started once the powder was mixed with liquid phase. After 1.5 minutes of mixing, gently touch the mixture with latex-gloved fingers. Observe the fibrous substance formed when fingers separate from the mixture surface. Repeat the touch test every 15 seconds until the fingers can detach cleanly. The initial observed time point was recorded as dough time. The bone cement was placed into the mold within 1 minute after reaching the dough state. An infrared thermal imager was used to continuously monitor temperature variations during polymerization, and real-time temperature data were recorded every 15 seconds. The time–versus–temperature curve was subsequently plotted. The setting time (*T*_set_) is the time when the temperature of the polymerizing mass is as follows: (*T*_max_ + *T*_amb_)/2*T*_max_ represents the maximum temperature (°C), and *T*_amb_ is the ambient temperature of 21 ± 1 °C. Four parallel samples were tested in each group.

To explore the practical performance of nisin-modified bone cement in joint prosthesis fixation, PALACOS® MV-N and J-MV-40-N cements were selected for joint prosthesis model experiments. After the bone cement was blended into the dough state, it was evenly coated on the surface of the knee prosthesis model. The spreading, void filling and interfacial adhesion properties of the material were observed.

### 2.9 Biocompatibility

#### 2.9.1 Determination of hemolysis rate

The experiment was conducted under constant temperature and humidity conditions, with an ambient temperature of 24 ± 1 °C and a relative humidity of 40% ± 5%. Briefly, 1.8 mL of bone cement extract, 1.8 mL of purified water, and 1.8 mL of normal saline were separately added into sterile centrifuge tubes, in which the purified water group served as the positive control and the normal saline group served as the negative control. Subsequently, 200 µL of 2% rabbit erythrocyte suspension was added to each tube, followed by incubation in a 37 °C, 5% CO_2_ incubator for 60 min. After incubation, all samples were centrifuged at 3000 rpm for 5 min. The occurrence of hemolysis in each group was preliminarily evaluated by observing the color and clarity of the supernatants, after which the absorbance (OD) values of the supernatants were measured at 545 nm using a microplate reader. Three parallel samples were prepared for each group (*n* = 3). According to the international standard method, the hemolysis ratio (HR) was calculated using the following formula:
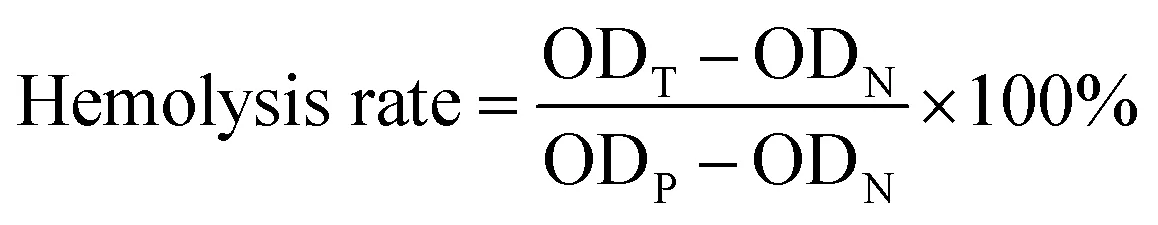
In the above equation, OD_T_ represents the optical density of the experimental group, OD_N_ represents the optical density of the negative control group (normal saline), and OD_P_ represents the optical density of the positive control group (distilled water).

#### 2.9.2 Determination of cytotoxicity

MC3T3-E1 cells were rapidly thawed in a 37 °C water bath and centrifuged at 1000 rpm for 4 min. After removal of the supernatant, the cells were resuspended in complete culture medium and cultured in a 37 °C, 5% CO_2_ incubator. MC3T3-E1 cells stably passaged to the third generation were used for subsequent experiments and maintained in complete medium specific for this cell line. For the experiments, MC3T3-E1 cells were seeded into 96-well plates at a density of 5 × 10^3^ cells per well, with 100 µL of complete medium added to each well. After 24 h of incubation, the cells had largely adhered to the plate surface. The original medium was then removed and replaced with 100 µL of experimental medium containing different bone cement extracts. The cells were co-cultured with the extracts for 1, 3, and 5 days, respectively. At each designated time point, CCK-8 working solution was added to each well at a final volume fraction of 10%. After an additional 120 min of incubation, the absorbance (OD) values were measured using a microplate reader at a detection wavelength of 450 nm and a reference wavelength of 630 nm. Three parallel wells were set for each group. The relative growth rate (RGR) of cells was calculated using the following formula:
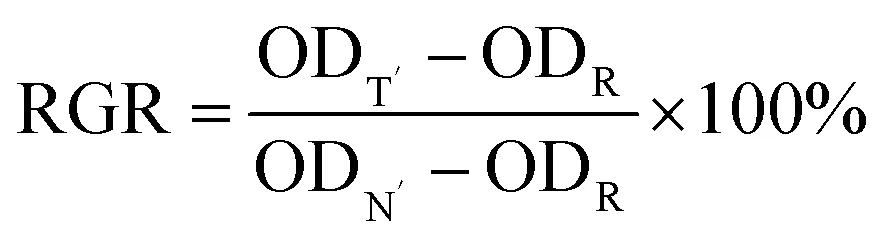
In the above equation, OD_T′_ represents the optical density of the experimental group, OD_N′_ represents the optical density of the control group (complete culture medium), and OD_R_ represents the optical density of the cell-free culture medium.

#### 2.9.3 Acute toxicity in mice

Thirty-five healthy SPF-grade C57BL/6 mice (body weight 18–22 g) were used in this study. All mice were randomly divided into seven groups (*n* = 5), including three bone cement control groups, three corresponding nisin-modified bone cement groups, and one saline negative control group. Each group received intraperitoneal injection at a dose of 50 mL kg^−1^ body weight. For the bone cement groups, mice were injected with the saline extracts of the corresponding materials, while the negative control group received an equal volume of normal saline. After administration, the general condition of the mice was monitored, including body temperature, respiration, food intake, and spontaneous activity. Body weight changes and abnormal reactions such as diarrhea and convulsions were recorded. At 72 h post-administration, the mice were euthanized by cervical dislocation, and liver and kidney tissues were harvested. The tissue samples were fixed in 4% paraformaldehyde. This protocol was approved by the Biomedical Ethics Committee of Hefei University of Technology (No. HFUT20241025-002).

### 2.10 Data statistics and analysis

Data collected in this study were statistically analyzed using SPSS 27.0 software, and the results were expressed as mean ± standard deviation. Independent-samples *t*-test was adopted to analyze compressive strength, MMA release, dough time, maximum temperature and setting time. One-way analysis of variance was used for antibacterial rate analysis. Repeated measures analysis of variance was applied to evaluate the relative growth rate.

## 3 Results and discussion

### 3.1 Mechanical properties

PMMA bone cement is widely used for bone defect filling and prosthesis fixation. Since the implanted material is required to bear mechanical loads *in vivo*, compressive strength is an important parameter for evaluating the mechanical performance of bone cement. Compressive strength testing was performed according to the ISO 5833 standard. Fig. 2a–c show the testing process, the morphology of the specimens before and after compression, and the stress–strain curves of the cements, respectively. As shown in Fig. 2d, the compressive strength of the three cements remained high after nisin incorporation, all exceeding the minimum standard requirement of 70 MPa. The strength of J-MV-40 cement was approximately 90 MPa and remained at 86 MPa after nisin incorporation, while PALACOS® MV and PALACOS® MV-N cement both exhibited strengths of approximately 85 MPa. No significant differences were observed before and after nisin incorporation in both groups (*p* > 0.05), indicating that the nisin-based technology had little effect on the compressive strength of J-MV-40 and PALACOS® MV cements. The mean compressive strength of MT-HA cement was 102 MPa, which was higher than that of J-MV-40 and PALACOS® MV bone cements, indicating its superior mechanical properties. After nisin incorporation, although the strength of MT-HA-N cement showed a slight but statistically significant decrease (*p* < 0.05), it remained at approximately 85 MPa, which was comparable to that of PALACOS® MV cement. Overall, all three clinical cements maintained good compressive strength after nisin incorporation and met the requirements of the ISO 5833 standard.

**Fig. 2:**
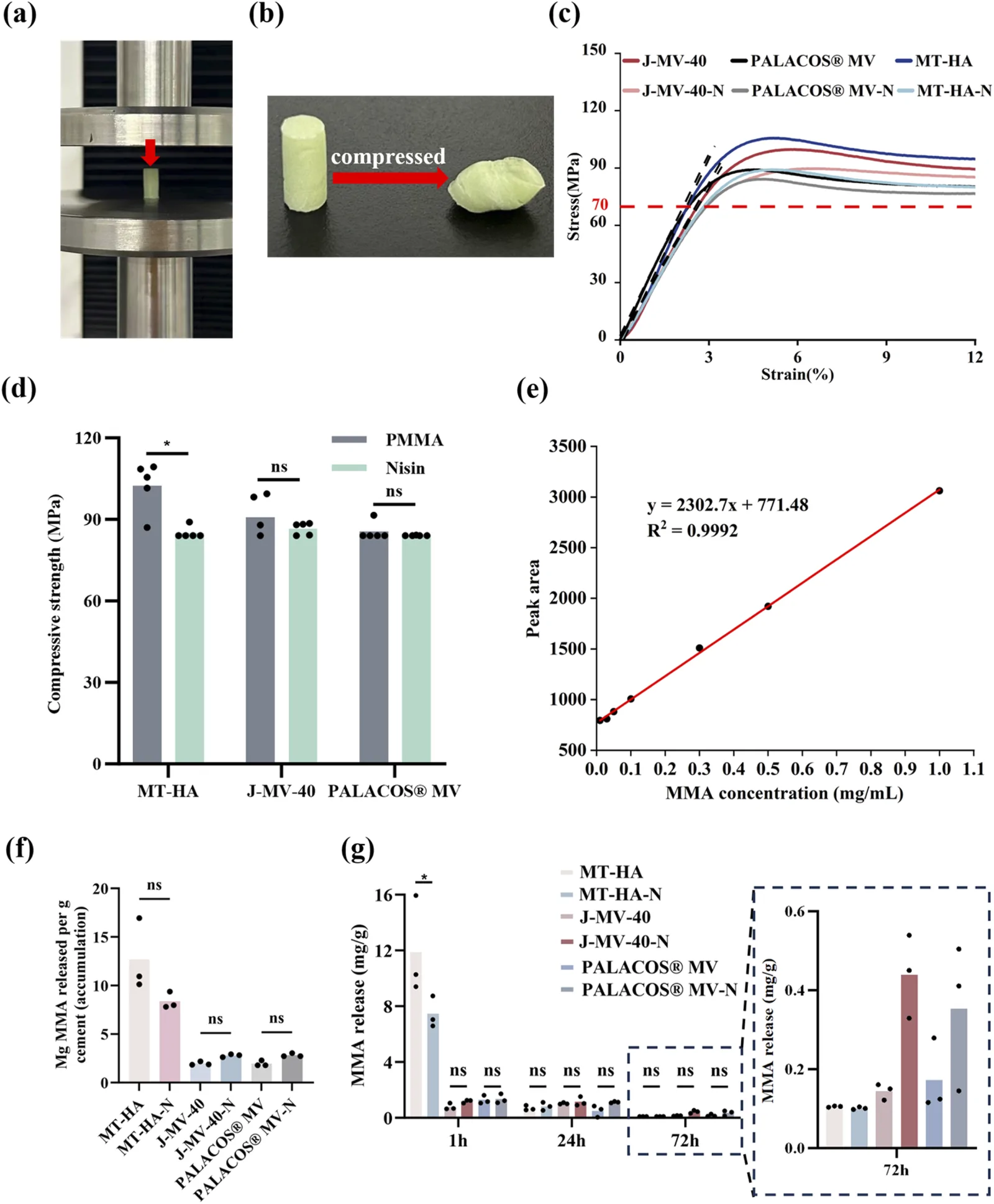
Mechanical properties and MMA release of PMMA bone cements before and after nisin modification. (a) Compressive testing process. (b) Morphology of bone cements before and after compression. (c) Stress–strain curves. (d) Compressive strength. (e) Calibration curve of MMA. (f) Cumulative MMA release. * indicates *p* < 0.05, and ns indicates no significant difference. (g) MMA release from bone cements at 1 h, 24 h and 72 h. The inset shows the enlarged view of MMA release at 72 h.

### 3.2 MMA monomer release

PMMA cement is prepared for clinical use by mixing prepolymerized PMMA with MMA monomer, followed by a polymerization reaction that leads to solidification. However, a small amount of residual MMA monomer is often retained in the cured cement, and its release after implantation may induce various complications. Therefore, from a clinical application perspective, it is necessary to further determine whether the incorporation of nisin increases the residual MMA monomer content. We further investigated the MMA release profiles of cement before and after the incorporation of nisin. Since different brands of cement differ in formulation composition and manufacturer-recommended powder-to-liquid ratios, this study does not aim to compare the absolute differences in MMA release among brands. Instead, it focuses on intra-brand changes before and after nisin addition to evaluate whether nisin affects MMA release behavior.

We established an MMA calibration curve, as shown in Fig. 2e. Excellent linearity was observed between MMA concentration and peak area, with a regression equation of *y* = 2302.7*x* + 771.48 and a coefficient of determination (*R*^2^) of 0.9992, indicating that this method is suitable for the quantitative determination of MMA concentration in the soaking solutions. The cumulative MMA release results are presented in Fig. 2f. As shown, no significant differences in cumulative MMA release were found between MT-HA and MT-HA-N cements, PALACOS® MV and PALACOS® MV-N cements, or J-MV-40 and J-MV-40-N cements (*p* > 0.05). These results indicate that the incorporation of nisin does not increase the cumulative MMA release of the three clinically used bone cements. These are encouraging results, providing strong evidence supporting the clinical adaptability of nisin-based technology. Notably, the MMA release of the MT-HA group was overall higher than that of the PALACOS® MV and J-MV-40 cements. This phenomenon may be related to the fact that the powder-to-liquid ratio recommended by the manufacturer for MT-HA cement was not strictly followed in this study. The recommended powder-to-liquid ratio for MT-HA cement is 2.5 g mL^−1^. In this work, we aimed to compare the performance differences of clinically used bone cements before and after the incorporation of nisin and to evaluate the clinical adaptability of nisin-based technology with commercially available bone cements. Therefore, a unified powder-to-liquid ratio was applied across all cements. Under these conditions, the liquid phase content of MT-HA cement was higher than the manufacturer-recommended formulation, which may account for its relatively higher early-stage MMA release. Further analysis of the single-time-point MMA release at different intervals is shown in Fig. 2g. The MMA release in all cements was primarily concentrated in the early stage. At 1 h, detectable amounts of MMA release were observed in all bone cements. After 24 h, the MMA release in all cements decreased markedly, and by 72 h, it further declined and remained at a low level. Notably, the MMA release of J-MV-40-N and PALACOS® MV-N cements at 72 h was slightly higher than that of the corresponding control cements. Except for the MT-HA cement at 1 h (*p* < 0.05), no significant changes in MMA release were observed after nisin incorporation for MT-HA at 24 h and 72 h, and for J-MV-40 and PALACOS® MV cements at all time points (*p* > 0.05). These findings are consistent with the cumulative release results, indicating that the nisin incorporated does not alter the release of residual monomer from the different brands of bone cement. Overall, the incorporation of nisin did not significantly alter the MMA release behavior of the three clinical cements (MT-HA, PALACOS® MV, and J-MV-40). These findings confirm that nisin modification does not increase the release risk of residual MMA monomer, and further suggest that nisin-based technology exhibits good compatibility with different clinically used cements.

### 3.3 SEM

Nisin-based antimicrobial peptide immobilization confers release profiles distinct from ALBC, as visualized by SEM of cross-sectional microstructures before and after aqueous immersion. We further characterized the cross-sectional morphology of PMMA cement, GS bone cement (ALBC), and nisin-modified cement by SEM to evaluate the morphological changes before and after immersion (Fig. 3, S1 and S2). PALACOS® MV cement was selected as the representative group in the main text (Fig. 3), while the SEM images of J-MV-40 and MT-HA are presented in Fig. S1 and S2, respectively. Owing to differences in the magnification used for capturing the overall cross-sectional morphology of each sample, the images from left to right correspond to magnifications of approximately 25–32×, 50×, and 100×, respectively, and the dashed boxes indicate the selected regions for local magnification. The fracture surfaces of both PMMA cement and nisin-modified cement appeared relatively smooth, with several round pores distributed on the surface, which were attributed to air bubbles introduced during the mixing process. SEM observation of the nisin-modified bone cement after immersion in water for 7 days revealed no obvious morphological changes compared with the non-immersed samples, consistent with our previous findings. In contrast, ALBC exhibited numerous pores on the fracture surface after immersion, which were likely formed by the dissolution and release of antibiotic clusters (white arrows). However, such characteristic pores were not observed in the nisin-modified bone cement, suggesting that nisin was stably immobilized within the cement matrix rather than released through cluster dissolution.

**Fig. 3:**
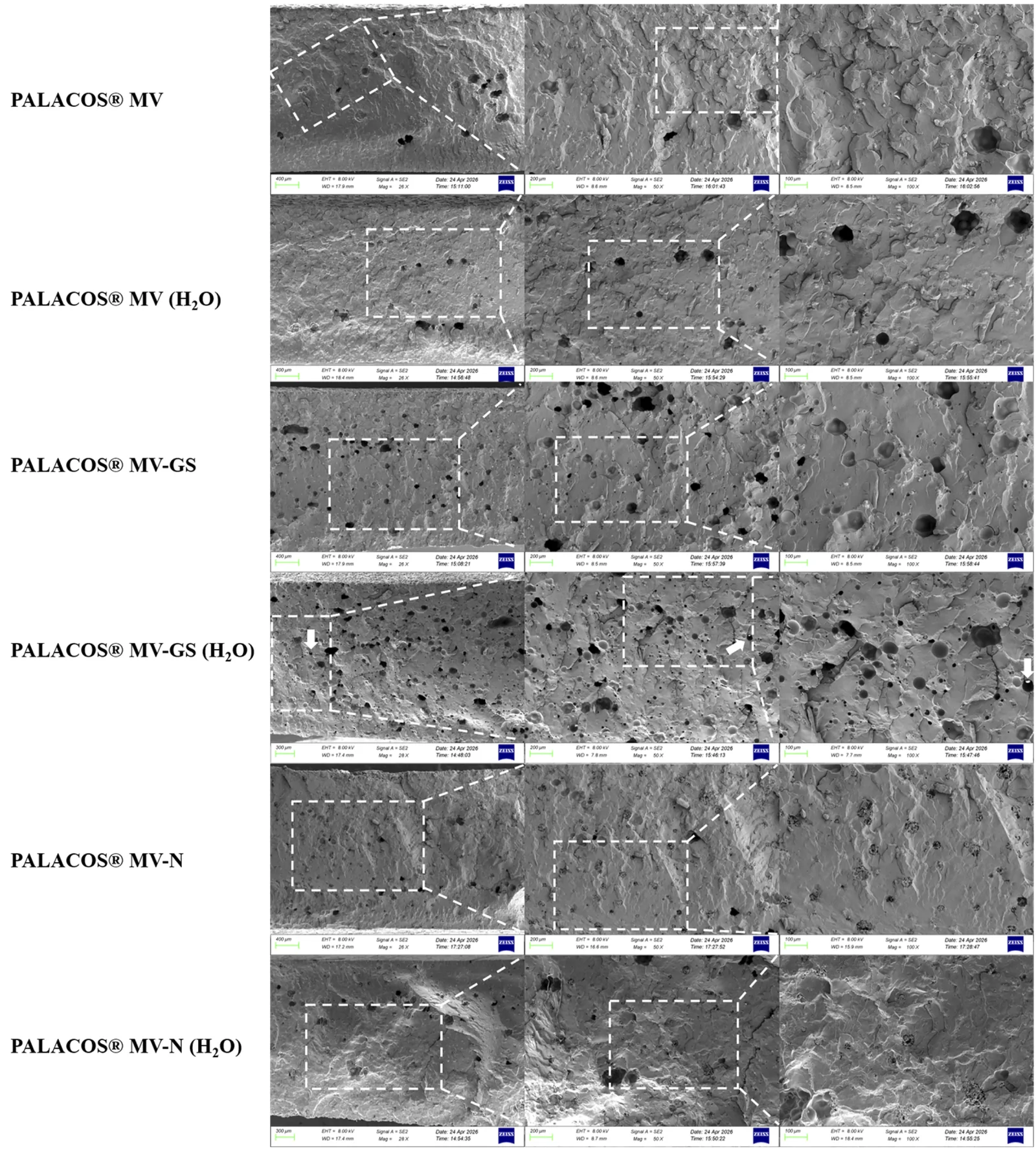
SEM images of the cross-sectional microstructures of PMMA, GS-loaded, and nisin-modified PALACOS® MV bone cements before and after immersion. From left to right, the magnifications are 26–28×, 50×, and 100×, respectively. The suffix (H_2_O) indicates the specimen after immersion in deionized water.

### 3.4 Antibacterial activity and the biophysical mechanisms of contact killing

*S. aureus* is the most common Gram-positive pathogen responsible for postoperative orthopedic infections and implant-associated infections. This bacterium readily adheres to biomaterial surfaces and can cause localized infections. In addition, it is capable of forming dense biofilm structures, which significantly reduce the efficacy of antimicrobial therapies. Therefore, *S. aureus* was selected as the model strain to systematically evaluate the antibacterial activity of nisin-modified cement. The classical agar diffusion assay was employed to assess the diffusion behavior of antibacterial substances. An inorganic CPC cement was included for comparison, where nisin was directly mixed into CPC-N cement. Clear inhibition zones formed around CPC-N, as CPC hardens without olefin polymerization and enables gradual nisin release, although zones were small owing to the low active nisin content in commercial preparations. Consistent with our prior work showing no inhibition zones for immobilized-nisin PMMA cement,^26^ with normal bacterial growth adjacent to samples, confirming successful surface immobilization rather than outward diffusion of nisin from the PMMA matrix (Fig. 4).

**Fig. 4:**
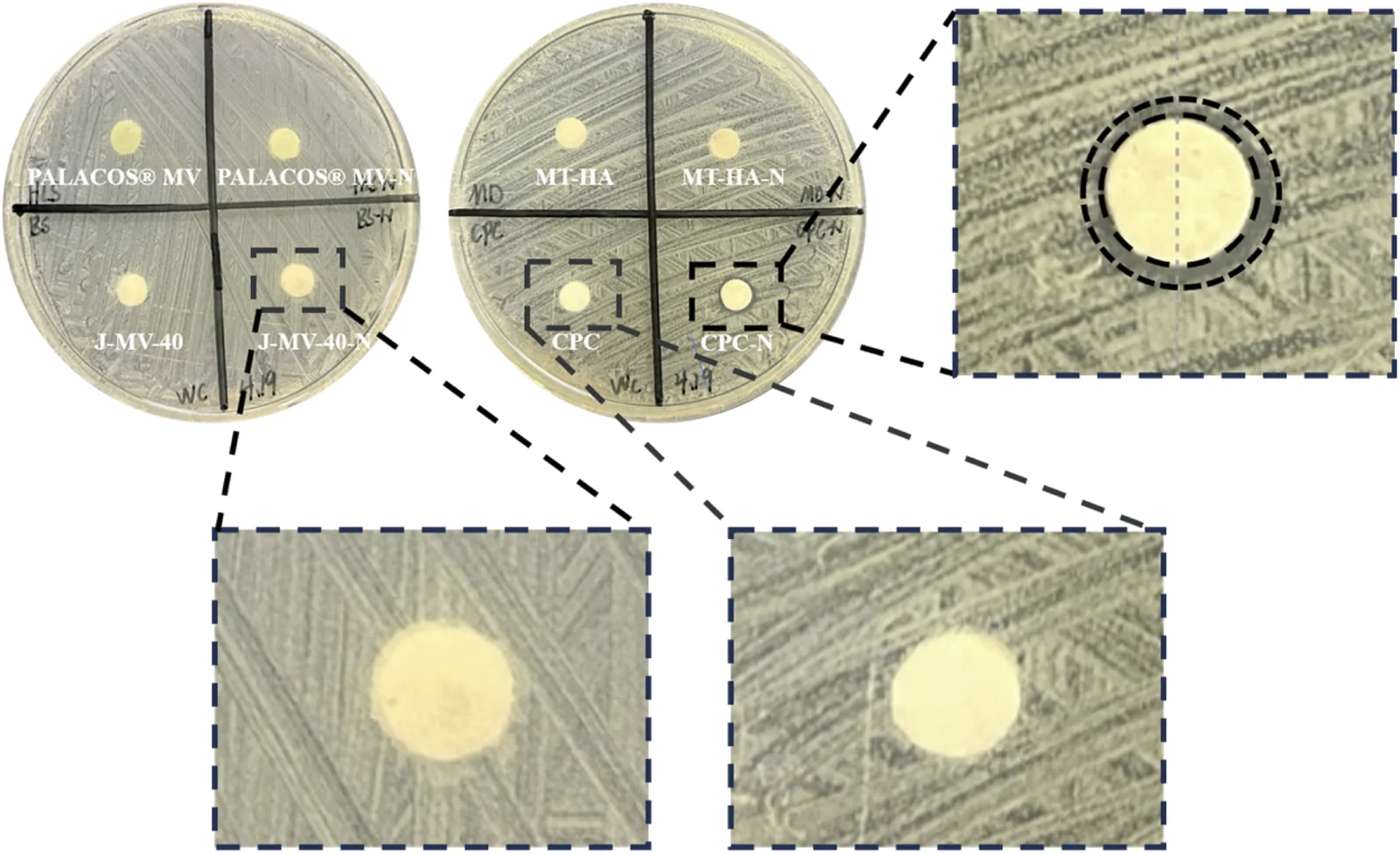
Inhibition zone assay of PMMA bone cement and CPC bone cement against *S. aureus*. Another set of parallel specimens is shown in Fig. S3.

With this encouraging result in hand, we further conducted surface antibacterial experiments (Fig. 5a). The plate culture results showed abundant bacterial colony growth in bare cements. In contrast, after the incorporation of nisin, the numbers of colonies on plates were markedly reduced (Fig. 5b). Colony counting demonstrated that all three nisin-modified clinical cements exhibited significant antibacterial activity, with antibacterial rates exceeding 94%. Notably, no bacterial colonies were detected in the J-MV-40-N group under the tested conditions (Fig. 5c). Analysis of viable bacterial counts expressed as log_10_(CFU per sample) showed a 1.24-log reduction for MT-HA-N and reductions of at least 2.84 and 2.17 log units for J-MV-40-N and PALACOS® MV-N, respectively, compared with their corresponding unmodified bone cements (Fig. S4). The dashed horizontal line in Fig. S4 indicates the operational limit of detection (LOD) at 3.800 log_10_ CFU per sample (corresponding to 6312.5 CFU per sample). In standardized bactericidal assays, a reduction of ≥3 log units (99.9%) in viable bacterial counts is commonly used as a reference criterion for bactericidal activity. In the present study, the antibacterial performance of nisin-modified bone cements was evaluated based on antibacterial rates and log reductions. Together with the absence of inhibition zones in the agar diffusion assays, these results further indicate that nisin-modified bone cement relies on a contact-killing mechanism based on biophysical interactions between immobilized AMP and bacterial cell membranes.

**Fig. 5:**
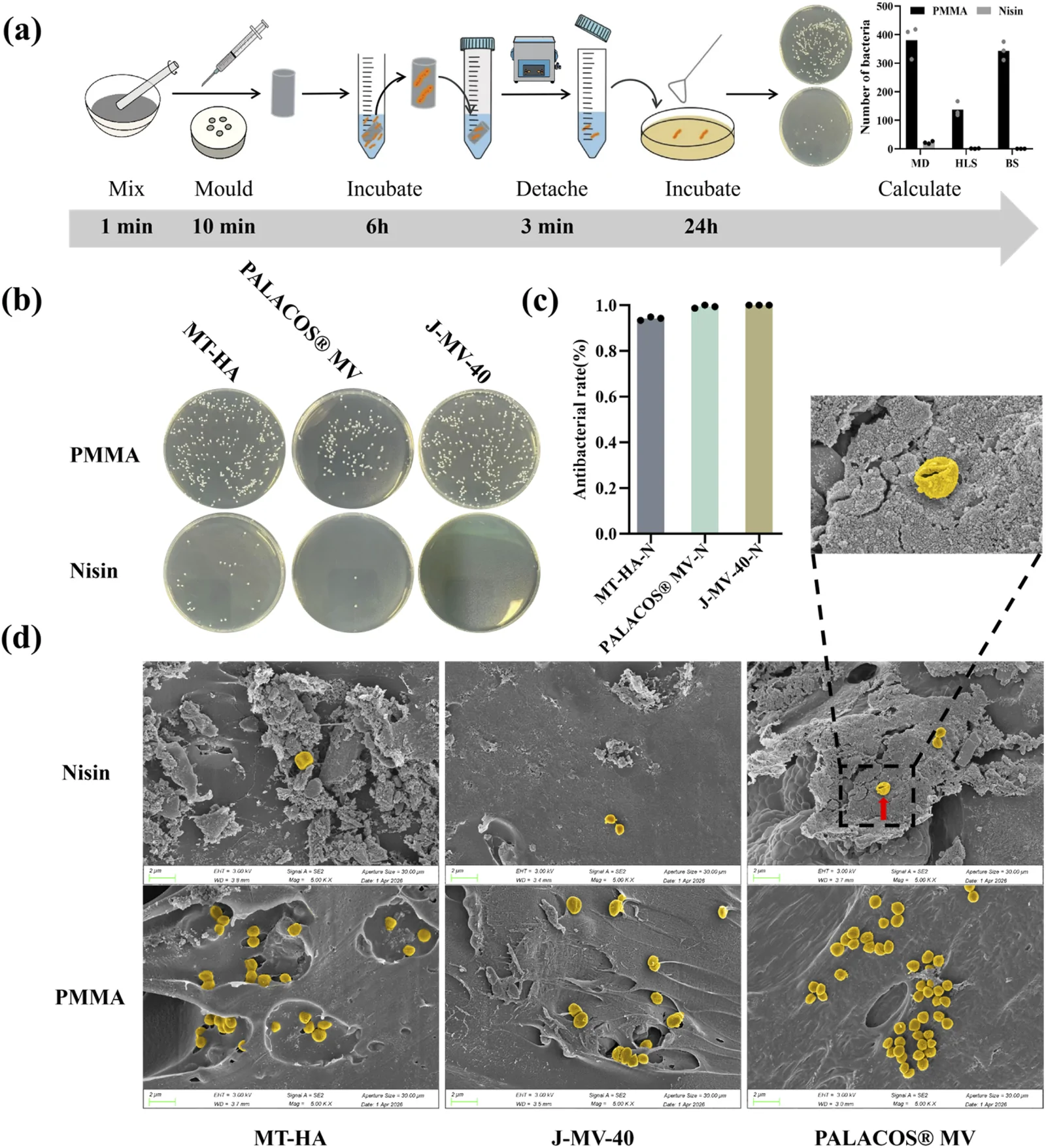
(a) Schematic diagram of the direct contact test (DCT) procedure. (b) The colony growth of *S. aureus* in culture dishes after treatment with PMMA bone cement and nisin-modified bone cement. (c) Antibacterial rate of nisin-modified bone cements. (d) SEM images of *S. aureus* on different bone cement surfaces. Bacteria are labeled in golden yellow. High-magnification images show morphological changes of bacteria on nisin-modified bone cement surfaces. Red arrows indicate damaged bacteria.

The antibacterial activity of nisin is synergistically mediated by its N-terminal domain, which recognizes and binds lipid II, and its C-terminal domain, which inserts into and disrupts the cell membrane. In the proposed immobilization strategy, Dha5 and Dha33 are potential reactive sites that may have different effects on antibacterial activity. Dha5 is located within ring A of the N-terminal lipid II-binding region, and its immobilization may introduce steric constraints that interfere with lipid II binding. Dha33 is located in the flexible C-terminal tail, and immobilization at this position may better preserve antibacterial activity by retaining the lipid II sequestration mechanism. However, whether immobilized nisin retains its characteristic pore-forming ability remains unclear. SEM observations showed extensive bacterial adhesion and aggregation on bare cements, whereas all three nisin-modified cements exhibited fewer adherent bacteria and reduced bacterial clustering (Fig. 5d). Higher-magnification SEM images further revealed bacterial wrinkling and collapse, suggesting bacterial structural damage. These findings support the antibacterial effectiveness of nisin-modified cements, although they do not directly establish the underlying molecular mechanisms. Future studies will investigate the effects of different immobilization sites on lipid II binding, membrane pore formation, and the overall antibacterial activity of nisin.

### 3.5 Handling performance

Handling performance are among the core indicators for evaluating the clinical applicability of bone cement. The ISO 5833 standards specify clear requirements for dough time, polymerization temperature, and setting time, all of which are closely associated with the surgeon's intraoperative manipulation and the overall surgical outcome. After mixing, the dough time and setting time together determine the clinical working window, thereby directly affecting material filling, shaping, and implant fixation during surgery. In addition, the exothermic reaction during cement polymerization and curing is an important indicator of clinical safety. After mixing the powder and liquid phases of cement, different handling stages can be identified according to its adhesion behavior toward latex gloves. When the cement still exhibits obvious adhesiveness and filament formation can be observed upon stretching with the glove, it is defined as the stringing phase (Fig. 6a). When the bone cement no longer shows obvious adhesion to the glove and is able to maintain an intact shape, it is defined as the dough phase^45^ (Fig. 6b). The dough time results are shown in Fig. 6c. Since MT-HA cement is a spinal-use formulation mainly used in spinal surgery, a relatively long clinical working window is generally required. Therefore, MT-HA cement exhibited the longest dough time, approximately 319 s. After the incorporation of nisin, the dough time was slightly prolonged to 337 s; however, no statistically significant difference was observed between MT-HA-N and MT-HA cement (*p* > 0.05). Both J-MV-40 and PALACOS® MV cements are joint-use formulations, whose clinical working windows are generally shorter than those of spinal-use bone cements. Specifically, the dough time of J-MV-40 cement was approximately 165 s, while that of PALACOS® MV cement was approximately 146 s. After nisin incorporation, the dough time of J-MV-40-N cement was slightly prolonged to 183 s, whereas the dough time of PALACOS® MV-N cement was slightly shortened to 142 s. None of these changes showed statistically significant differences compared with their corresponding control cements (*p* > 0.05). Overall, the incorporation of nisin did not significantly alter the dough time of the three clinical cements. Subsequently, the dynamic temperature changes during the polymerization process of the cement were monitored in real time, and temperature–time curves were plotted. Since the temperature–time curves of all cements exhibited similar trends, only one representative temperature–time curve of PALACOS® MV-N cement is shown in Fig. 6d, while the complete temperature variation data for all cements are provided in SI1. After the bone cement entered the dough phase, the temperature remained relatively stable initially. As the internal polymerization reaction proceeded, the temperature of the system rapidly increased, gradually decreased after reaching its peak value. Based on the temperature–time curves, maximum polymerization temperature (*T*_max_) of each cement was determined. As shown in Fig. 6e, the maximum polymerization temperature results demonstrated that the exothermic temperature of J-MV-40 cement remained essentially unchanged after the incorporation of nisin (*p* > 0.05), maintaining a stable range of 58–59 °C. For PALACOS® MV cement, the peak temperature increased from 53 °C to 62 °C after the addition of nisin; however, the difference was not statistically significant (*p* > 0.05). Notably, after nisin incorporation, the maximum polymerization temperature of MT-HA cement significantly decreased from 59 °C to 50 °C (*p* < 0.05). The setting time (*T*_set_) was defined as the time at which the temperature of the polymerizing material reached (*T*_max_ + *T*_amb_)/2, where *T*_amb_ represents the ambient temperature of 21 ± 1 °C. As shown in Fig. 6f, after the incorporation of nisin, the setting time of MT-HA bone cement was prolonged from approximately 9 min to 11–12 min, and a statistically significant difference was observed between MT-HA and MT-HA-N (*p* < 0.05). In contrast, the setting time of J-MV-40 cement remained stable at 8–9 min before and after nisin incorporation, with no statistically significant difference observed (*p* > 0.05). Similarly, the setting time of PALACOS® MV cement remained within 7–8 min, and no significant difference was detected after the addition of nisin (*p* > 0.05). Overall, the influence of nisin on the polymerization exothermic temperature varied among different brands of bone cement, although the peak temperatures of all cements remained within clinically acceptable ranges. These findings suggest that the nisin-based technology did not impair the handling properties of the three commercially available bone cements. It should be noted that, in order to strictly control the experimental variables, all cements in the present study were prepared using a unified powder-to-liquid ratio rather than fully following the manufacturer-recommended formulations. The use of a unified powder-to-liquid ratio may also account for the slightly longer dough time of MT-HA cement compared with conventional industry standards.

**Fig. 6:**
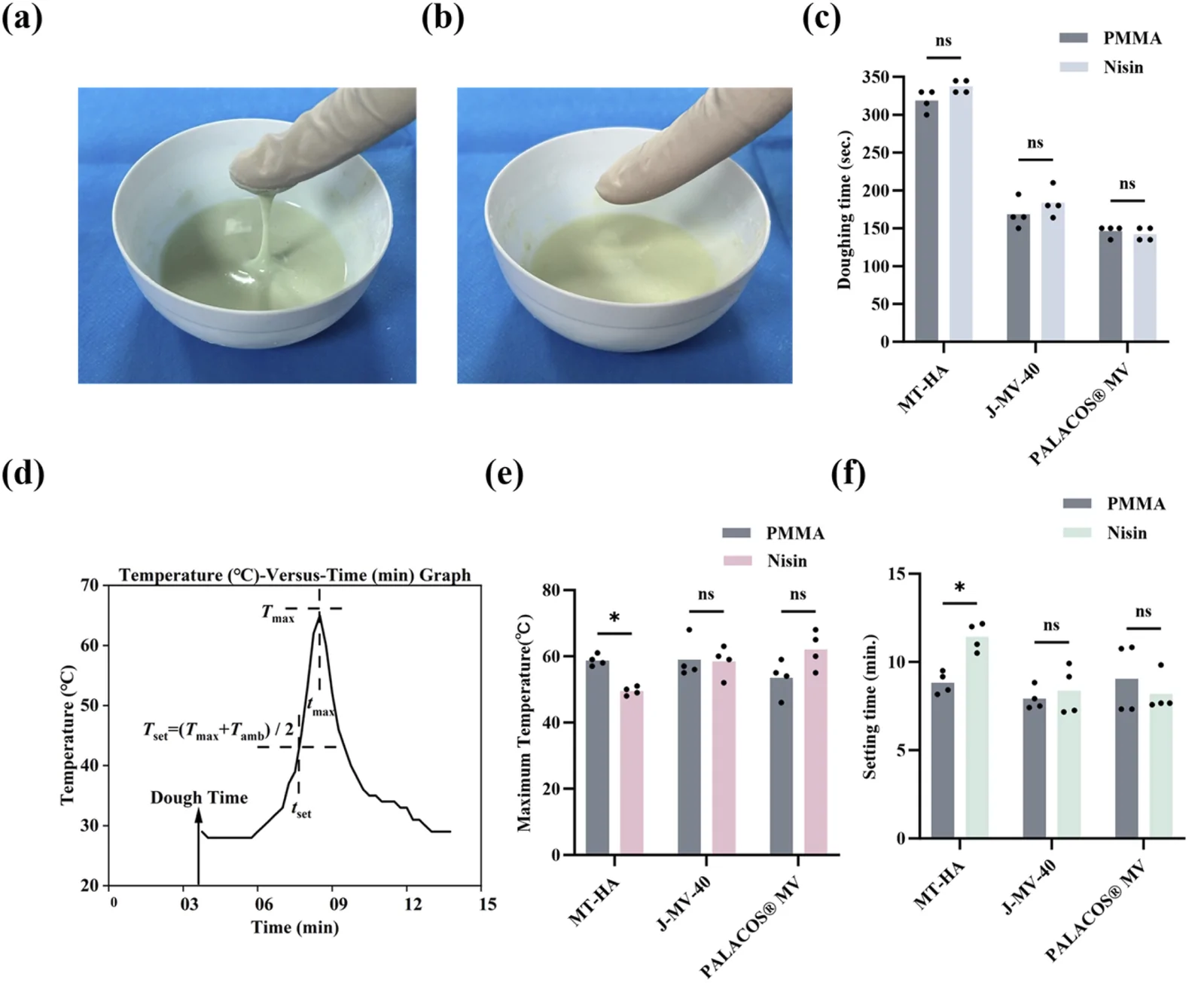
Handling and curing behavior of bone cements. (a) Stringing stage. (b) Dough stage. (c) Doughing time. (d) Temperature–time curve. (e) Maximum temperature. (f) Setting time. * indicates *p* < 0.05, and ns indicates no significant difference.

To further enhance the clinical relevance of this study, the handling properties of cements were re-evaluated using the manufacturer-recommended powder-to-liquid ratios (Fig. S5). The results showed that, under the manufacturer-recommended mixing conditions, the dough time of the PALACOS® MV cement was shortened from 139 s to 98 s after the incorporation of nisin (*p* < 0.05), whereas no statistically significant differences were observed in any other handling parameters between the remaining cements and their corresponding controls (*p* > 0.05). These findings indicate that, even under the manufacturer-recommended formulations, the incorporation of nisin did not alter the clinical handling properties of the three bone cements.

### 3.6 Translational applications

PMMA bone cement is widely used in spinal procedures such as percutaneous vertebroplasty and percutaneous kyphoplasty. In these procedures, cement must be injected into the vertebral body through a working channel; therefore, the injectability, anti-leakage capability, and filling performance of the cement are critical for clinical application. Accordingly, the present study further investigated the effects of nisin incorporation on the injectability, intravertebral filling performance, and leakage behavior of bone cement. Among the three clinically used PMMA cements, MT-HA cement is mainly used in spinal surgery; therefore, MT-HA-N was selected for validation in this section. From an application-oriented perspective, a 3D-printed human vertebral model and a porcine vertebral model were employed to evaluate the relevant properties of the bone cement. As shown in Fig. 7a, the nisin-modified bone cement could be continuously extruded during injection, indicating good injectability. Subsequently, porous sponges were implanted into the 3D-printed vertebral model to simulate the porous structure formed after vertebral compression. Nisin-modified cement was then injected through a syringe to evaluate its filling and anti-leakage performance. After injection into the vertebral model, removal of the surface foam revealed a relatively regular elliptical distribution of the bone cement within the model, indicating good filling capability and a low risk of leakage (Fig. 7b). In addition, a transpedicular working channel was established in porcine vertebrae, and the distribution of the bone cement was observed using reconstructed computed tomography (CT) imaging (Fig. 7c). The results demonstrated that the bone cement was uniformly distributed within the channel, without obvious leakage. The *ex vivo* vertebral injection experiments further confirmed that the nisin-modified cement possessed good injectability and filling performance. Taken together, these findings indicate that the incorporation of nisin did not affect the injection procedure or intravertebral filling performance of MT-HA cement, and that the modified bone cement still retained good injectability, filling ability, and anti-leakage capability.

**Fig. 7:**
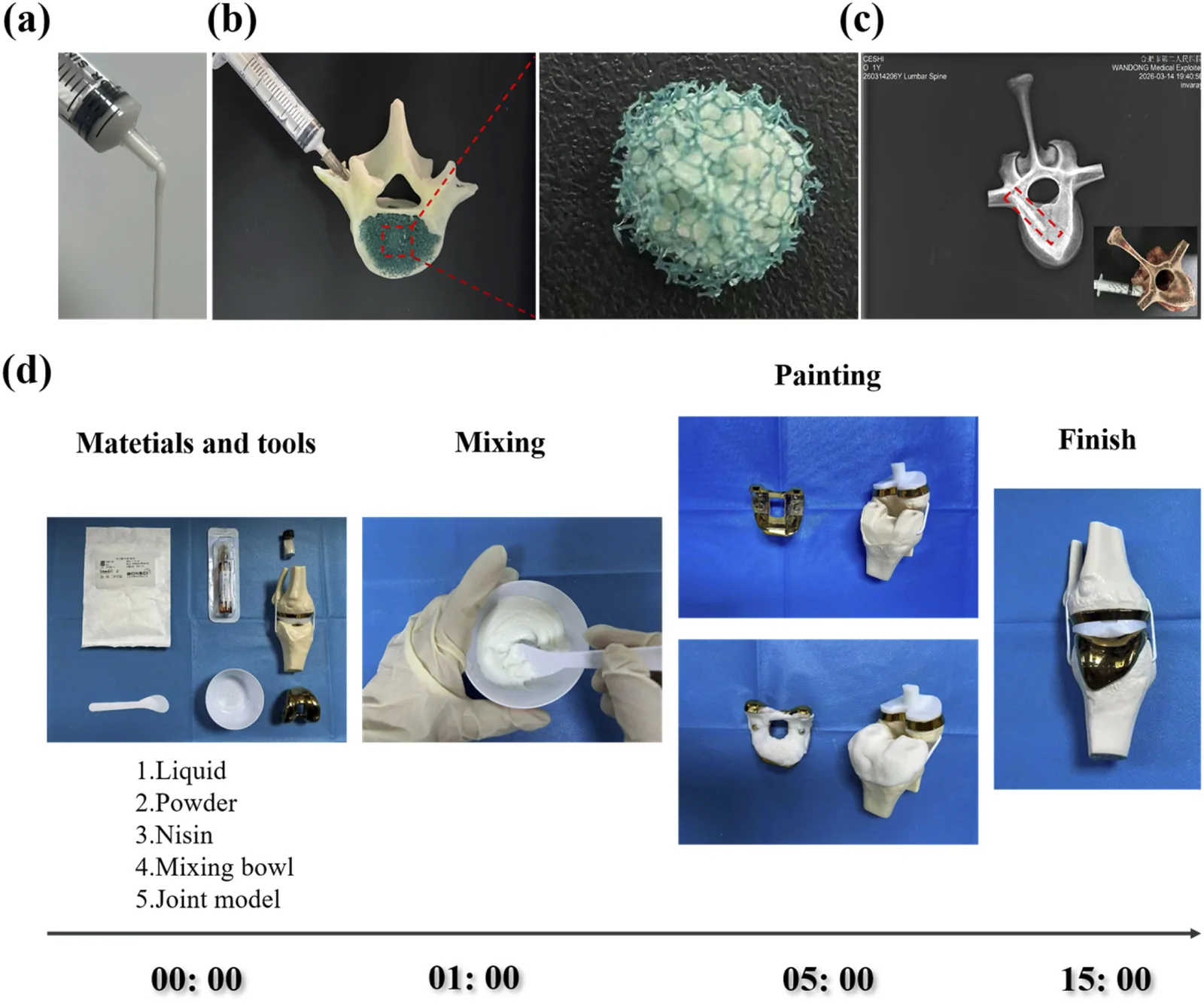
(a) Injectability of nisin-modified bone cement. (b) Filling behavior of nisin-modified bone cement in a 3D-printed human vertebral body model. (c) X-ray image of the vertebral model after cement injection. (d) Operating room demonstration of nisin-modified J-MV-40 bone cement.

To further investigate the clinical relevance of the nisin-based technology, a simulated joint prosthesis replacement experiment was performed under operating room conditions. Among the three bone cements evaluated, PALACOS® MV and J-MV-40 cements are clinically used for joint replacement surgery; therefore, these two cements were selected for the simulation experiments. J-MV-40-N cement was chosen as the representative to demonstrate the application of the nisin-based technology during joint prosthesis replacement (Fig. 7d), while the simulated surgical procedure using PALACOS® MV-N bone cement is provided in the SI (Fig. S6). The materials prepared for the experiment included nisin, bone cement powder, liquid monomer, mixing containers, stirring rods, a knee joint model, and a joint prosthesis. During the procedure, the cement powder and nisin were first placed into a mixing bowl, followed by the addition of the liquid monomer phase and continuous stirring for 1 min. After the bone cement reached the dough phase, it was evenly applied to the surface of the joint and the contact surface of the joint prosthesis. The prosthesis was then implanted and fixed in place, and the excess extruded cement was removed to complete the simulated prosthesis replacement procedure. In the simulated setting, the nisin-modified cements could be prepared and applied following the described mixing and fixation procedures. Together with the handling property results, these observations support the feasibility of incorporating the nisin-based technology into the simulated prosthesis fixation procedure. Future usability studies under clinically relevant conditions will further evaluate its applicability in routine surgical practice and provide additional evidence to support its potential clinical translation.

### 3.7 *In vitro* biocompatibility (hemolysis and cytotoxicity)

Biocompatibility is a fundamental requirement for biomedical materials. Therefore, hemolysis and cytotoxicity assays were performed to evaluate the influence of the nisin-based technology on the biocompatibility of three clinically used cements. Fig. 8a shows the hemolysis results of each cement, while Fig. 8b presents the corresponding quantitative analysis of hemolysis ratios. The results demonstrated that the hemolysis ratios of all cements and their corresponding nisin-modified cements were below 5%. Specifically, the hemolysis ratio of J-MV-40 cement was approximately 1% and increased to 2.5% after nisin incorporation, although the difference was not statistically significant (*p* > 0.05); the PALACOS® MV and PALACOS® MV-N cements both maintained low hemolysis ratios without significant differences (*p* > 0.05). Unlike the other two groups, the hemolysis ratio of MT-HA cement significantly decreased after the addition of nisin, and the difference was statistically significant (*p* < 0.05). Overall, the nisin-based technology did not significantly increase the hemolysis ratios of the three clinically used PMMA bone cements under the tested conditions, while MT-HA-N cement exhibited a significantly lower hemolysis ratio than unmodified MT-HA. Furthermore, the effects of the three clinically used PMMA bone cements and their modified cements on cell proliferation were evaluated using the cell counting kit-8 (CCK-8) assay, and the results are shown in Fig. 8c. The relative growth rates (RGR) of all groups after 1, 3, and 5 days of culture were higher than 75%, meeting the requirements of the GB/T 16 886 standard for cytotoxicity evaluation of medical devices, indicating that none of the materials exhibited obvious cytotoxicity. After 1 day of culture, the RGR of all groups remained close to 100%. After 3 and 5 days of culture, although slight fluctuations were observed among the groups, the overall RGR remained at relatively high levels. No statistically significant differences in RGR were detected before and after modification within the same brand of cement (*p* > 0.05). These results confirmed that the incorporation of nisin did not inhibit the growth of MC3T3-E1 cells, and all three nisin-modified PMMA bone cements exhibited good cytocompatibility.

**Fig. 8:**
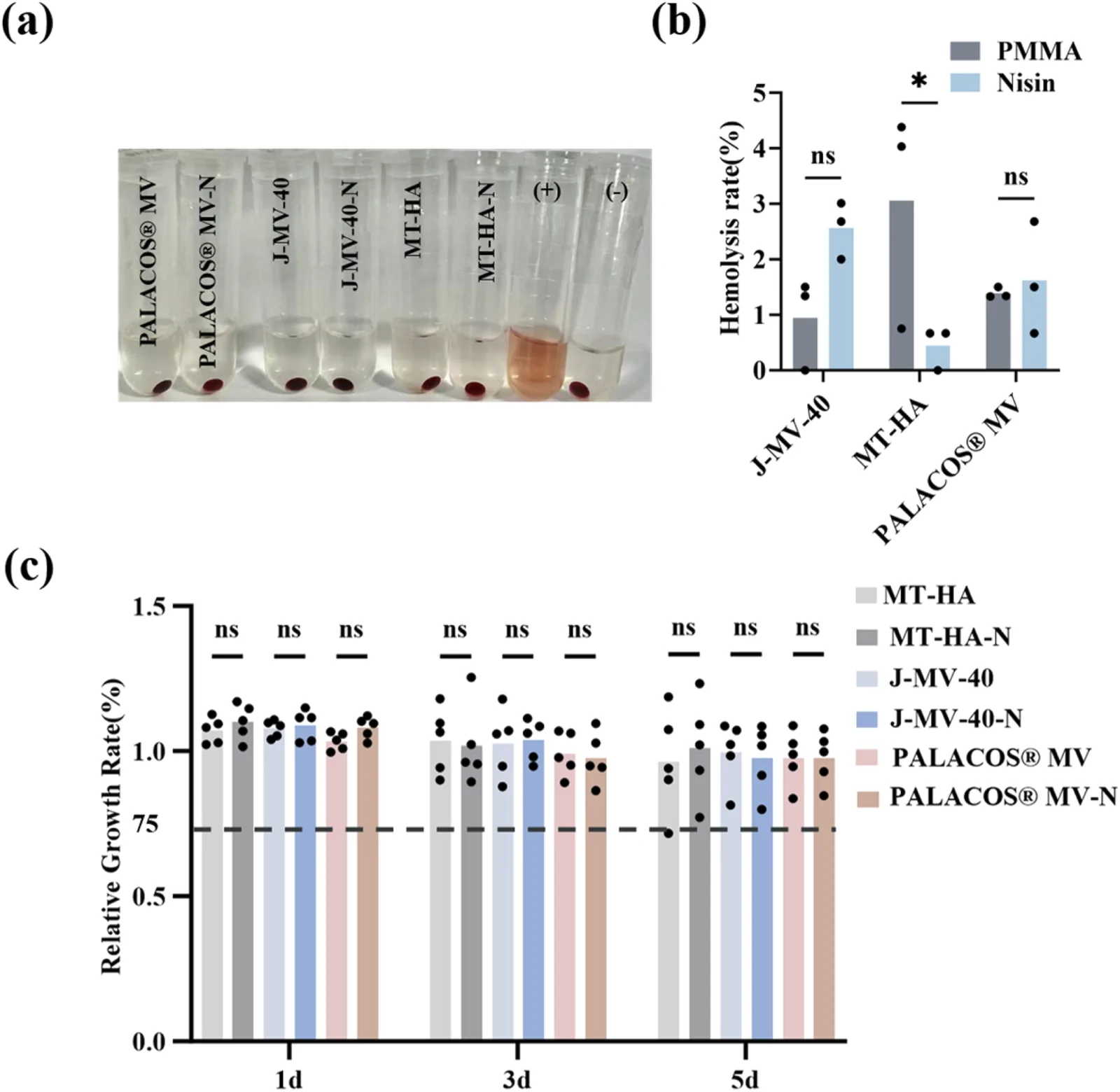
Hemocompatibility and cytocompatibility of PMMA and nisin bone cements. (a) Visual images of the hemolysis test. (b) Hemolysis rates of different bone cements. (c) RGR after incubation with different bone cement extracts for 1, 3, and 5 days. * indicates *p* < 0.05, and ns indicates no significant difference.

### 3.8 Acute toxicity

To evaluate the *in vivo* safety of the nisin-modified bone cement extracts, a mouse intraperitoneal injection model was employed. After administration, all mice exhibited good general condition, without obvious adverse manifestations such as reduced activity, abnormal food intake, diarrhea, or convulsions. No significant body weight loss was observed in any group during the observation period (Fig. S7). On day 3 after treatment, the mice were euthanized, and liver and kidney tissues were collected for H & E histological staining. As shown in Fig. 9a, the liver tissues of all groups maintained intact histological structures, with regularly arranged hepatocytes and clear hepatic lobule architecture, without obvious tissue necrosis or inflammatory cell infiltration. Similarly, the kidney tissues retained normal morphology (Fig. 9b), with intact glomerular structures and well-arranged renal tubules, and no evidence of glomerular atrophy, tubular necrosis, or significant inflammatory responses was observed. Compared with the saline group, the extracts of the three PMMA cements and their corresponding nisin-modified cements did not induce any obvious histopathological changes in the liver or kidney tissues. These results suggest that the extracts of nisin-modified bone cements caused no apparent acute histopathological changes in the liver or kidneys and exhibited favorable short-term *in vivo* tolerability.

**Fig. 9:**
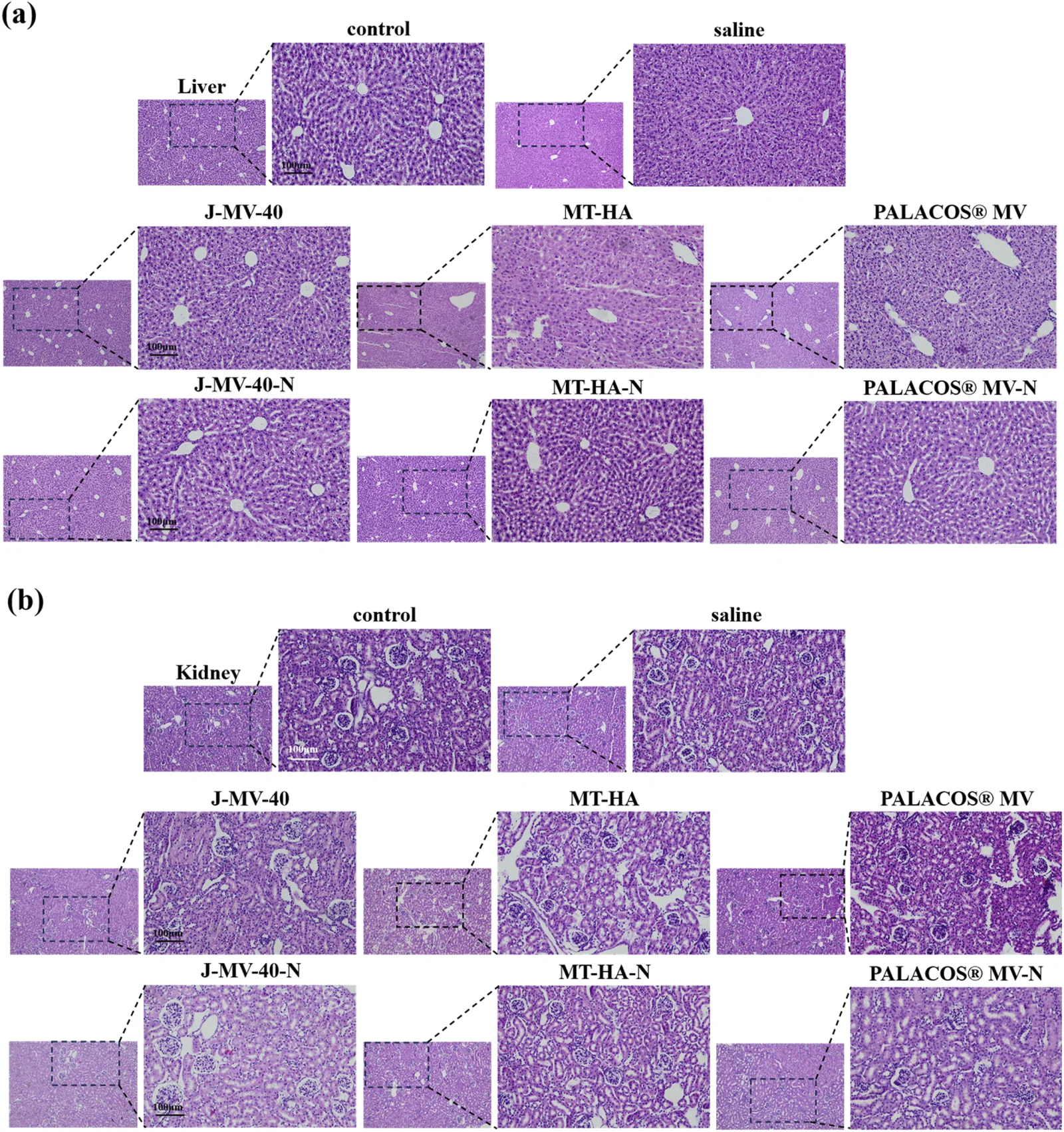
(a) The liver sections of mice with bare cement and nisin-modified cement. (b) The kidney sections of mice with bare cement and nisin-modified cement.

## 4 Limitations

The current safety evaluation was mainly based on short-term *in vitro* assays and limited animal histological observations, which cannot fully establish the long-term safety of nisin-modified commercial PMMA bone cements. More comprehensive and long-term preclinical studies are therefore needed. Furthermore, although our previous and present studies have provided indirect evidence supporting the proposed covalent immobilization mechanism, direct chemical evidence confirming the formation of covalent bonds between nisin and the PMMA matrix remains lacking. Further molecular-level characterization is needed to conclusively establish the immobilization mechanism. In addition, the nisin-based modification strategy remains at an early stage of clinical translation, as practical factors such as mixing procedures, intraoperative contaminants (*e.g.*, blood, bone fragments, and saline), and minor variations in component ratios were not considered in this study. Further academic–industrial collaboration is also essential to advance translational development of this nisin-based strategy.

## 5 Conclusions

This study systematically validates the universal compatibility of the one-step nisin-based antimicrobial peptide immobilization technique with three commercial clinical PMMA bone cements, addressing the critical bottleneck for its clinical translation. Nisin covalent immobilization does not compromise the core performance of clinical bone cements: all modified cements meet the ISO 5833 compressive strength standard, show no significant changes in residual MMA release profiles, and exert significant bactericidal activity against *S. aureus via* a contact-killing mechanism, with antibacterial rates exceeding 94% across all formulations and reaching nearly 100% for one commercial cement. Importantly, the modified cements retain favorable clinical handling properties, injectability, and *in vitro*/*in vivo* biocompatibility, remaining fully compatible with standard surgical workflows and operable by general clinicians without additional specialized equipment. This convenient, low-cost, and safe strategy provides a robust platform for the clinical translation of high-performance anti-infective bone cements. We are committed to sharing this protocol free of charge with frontline clinicians and researchers worldwide, with the ultimate goal of reducing the global burden of orthopedic implant infections and equitably benefiting patients in low- and middle-income countries and regions affected by prolonged armed conflict. Future work will optimize product-specific formulations, evaluate long-term *in vivo* antibacterial efficacy and osseointegration, and advance academic-industrial collaboration to accelerate global clinical implementation.

## Author contributions

Conception and design of the research: Yang Xu, Jianjun Chu, Fang He, Yiming Li. Acquisition of data: Chen Wang, Hao Lin, Bo Lu. Analysis and interpretation of the data: Chen Wang, Yang Xu, Bo Lu, Zhaoyang Li. Statistical analysis: Chen Wang, Hao Lin. Obtaining financing: Yang Xu, Fang He, Jianjun Chu. Writing of the manuscript: Chen Wang, Yang Xu. Critical revision of the manuscript for intellectual content: Yang Xu, Jianjun Chu, Fang He, Zhaoyang Li.

## Conflicts of interest

The authors declare that they have no known competing financial interests or personal relationships that could have appeared to influence the work reported in this paper.

## Supplementary Material

RA-OLF-D6RA08377C-s001

RA-OLF-D6RA08377C-s002

RA-OLF-D6RA08377C-s003

## Data Availability

The data used to support the findings of this study are available from the corresponding author upon request. Supplementary information is available. See DOI: https://doi.org/10.1039/d6ra08377c.

## References

[cit1] Lin H., Gao Z., Shan T., Asilebieke A., Guo R., Kan Y.-C., Li C., Xu Y., Chu J.-J. (2024). A Review on the Promising Antibacterial Agents in Bone Cement—From Past to Current Insights. J. Orthop. Surg. Res..

[cit2] Agni N. R., Costa M. L., Achten J., Peckham N., Dutton S. J., Png M. E., Reed M. R. (2023). WHiTE 8 Investigators. High-Dose Dual-Antibiotic Loaded Cement for Hip Hemiarthroplasty in the UK (WHiTE 8): A Randomised Controlled Trial. Lancet.

[cit3] Al Thaher Y., Perni S., Prokopovich P. (2017). Nano-Carrier Based Drug Delivery Systems for Sustained Antimicrobial Agent Release from Orthopaedic Cementous Material. Adv. Colloid Interface Sci..

[cit4] Kurtz S. M., Lau E. C., Son M.-S., Chang E. T., Zimmerli W., Parvizi J. (2018). Are We Winning or Losing the Battle with Periprosthetic Joint Infection: Trends in Periprosthetic Joint Infection and Mortality Risk for the Medicare Population. J. Arthroplasty.

[cit5] Xu Y.-M., Peng H.-M., Feng B., Weng X.-S. (2020). Progress of Antibiotic-Loaded Bone Cement in Joint Arthroplasty. Chin. Med. J..

[cit6] Sloan M., Premkumar A., Sheth N. P. (2018). Projected Volume of Primary Total Joint Arthroplasty in the US, 2014 to 2030. J. Bone Jt. Surg., Am..

[cit7] Ghosh S. , Sinha M., Samanta R., Sadhasivam S., Bhattacharyya A., Nandy A., Saini S., Tandon N., Singh H., Gupta S., Chauhan A., Aavula K. K., Varghese S. S., Shi P., Ghosh S., Garg M. K., Saha T., Padhye A., Ghosh S., Jang H. L., and Sengupta S., A Potent Antibiotic-Loaded Bone-Cement Implant against Staphylococcal Bone Infections, Nat. Biomed. Eng., 2022, 6, 1180 1195, https://search.crossref.org/search/works?q=A+potent+antibiotic-loaded+bone-cement+implant+against+staphylococcal+bone+infections&from_ui=yes 10.1038/s41551-022-00950-x PMC10101771 36229662

[cit8] Peddada K. V., Welcome B. M., Parker M. C., Delman C. M., Holland C. T., Giordani M., Meehan J. P., Lum Z. C. (2023). Survivorship and Etiologies of Failure in Single-Stage Revision Arthroplasty for Periprosthetic Joint Infection: A Meta-Analysis. J. Am. Acad. Orthop. Surg. Glob. Res. Rev..

[cit9] Kubista B., Hartzler R. U., Wood C. M., Osmon D. R., Hanssen A. D., Lewallen D. G. (2012). Reinfection after Two-Stage Revision for Periprosthetic Infection of Total Knee Arthroplasty. Int. Orthop..

[cit10] Lentino J. R. (2003). Prosthetic Joint Infections: Bane of Orthopedists, Challenge for Infectious Disease Specialists. Clin. Infect. Dis..

[cit11] Chen L., Tang Y., Zhao K., Zha X., Liu J., Bai H., Wu Z. (2019). Fabrication of the Antibiotic-Releasing Gelatin/PMMA Bone Cement. Colloids Surf., B.

[cit12] Huang M.-H., Chen Y.-H., Pu C.-C., Ding Y.-J., Hsu Y.-P., Yang N.-P., Siu L. K. (2025). Entrapping imipenem into poly(methyl methacrylate) assemblies by microfluidic process retains the activity against prosthetic joint infections following thermal treatment. Colloids Surf., B.

[cit13] Czuban M., Wulsten D., Wang L., Di Luca M., Trampuz A. (2019). Release of different amphotericin B formulations from PMMA bone cements and their activity against Candida biofilm. Colloids Surf., B.

[cit14] Mistry S., Roy R., Jha A. K., Pandit N., Das S., Burman S., Joy M. (2022). Treatment of long bone infection by a biodegradable bone cement releasing antibiotics in human. J. Controlled Release.

[cit15] Leta T. H., Fenstad A. M., Lygre S. H. L., Lie S. A., Lindberg-Larsen M., Pedersen A. B., W-Dahl A., Rolfson O., Bülow E., Ashforth J. A., Van Steenbergen L. N., Nelissen R. G. H. H., Harries D., De Steiger R., Lutro O., Hakulinen E., Mäkelä K., Willis J., Wyatt M., Frampton C., Grimberg A., Steinbrück A., Wu Y., Armaroli C., Molinari M., Picus R., Mullen K., Illgen R., Stoica I. C., Vorovenci A. E., Dragomirescu D., Dale H., Brand C., Christen B., Shapiro J., Wilkinson J. M., Armstrong R., Wooster K., Hallan G., Gjertsen J.-E., Chang R. N., Prentice H. A., Paxton E. W., Furnes O. (2023). The use of antibiotic-loaded bone cement and systemic antibiotic prophylactic use in 2,971,357 primary total knee arthroplasties from 2010 to 2020: an international register-based observational study among countries in Africa, Europe, North America, and Oceania. Acta Orthop..

[cit16] Jiang J., Wang J., Fan P., Zhao Z., Deng H., Li J., Wang Y., Wang Y. (2025). Biomaterial-based strategies for bone cement: modulating the bone microenvironment and promoting regeneration. J. Nanobiotechnol..

[cit17] Tootsi K., Heesen V., Lohrengel M., Enz A. E., Illiger S., Mittelmeier W., Lohmann C. H. (2022). The use of antibiotic-loaded bone cement does not increase antibiotic resistance after primary total joint arthroplasty. Knee Surg. Sports Traumatol. Arthrosc..

[cit18] Sivabalan P., Samways D. S. K., Yuya P. A., Shipp D. A. (2026). Polymer-based Bone Cements: Chemistry, Physicochemical Properties, and Biological Considerations. ACS Appl. Polym. Mater..

[cit19] Leta T. H., Lie S. A., Fenstad A. M., Lygre S. H. L., Lindberg-Larsen M., Pedersen A. B., W-Dahl A., Rolfson O., Bülow E., van Steenbergen L. N., Nelissen R. G. H. H., Harries D., de Steiger R., Lutro O., Mäkelä K., Venäläinen M. S., Willis J., Wyatt M., Frampton C., Grimberg A., Steinbrück A., Wu Y., Armaroli C., Gentilini M. A., Picus R., Bonetti M., Dragosloveanu S., Vorovenci A. E., Dragomirescu D., Dale H., Brand C., Christen B., Shapiro J., Wilkinson J. M., Armstrong R., Wooster K., Hallan G., Gjertsen J.-E., Chang R. N., Prentice H. A., Sedrakyan A., Paxton E. W., Furnes O. (2024). Periprosthetic Joint Infection After Total Knee Arthroplasty With or Without Antibiotic Bone Cement. JAMA Netw. Open.

[cit20] Suhardi V. J., Bichara D. A., Kwok S. J. J., Freiberg A. A., Rubash H., Malchau H., Yun S. H., Muratoglu O. K., Oral E. (2017). A fully functional drug-eluting joint implant. Nat. Biomed. Eng..

[cit21] Wu Y., Lin Y., Cong Z., Chen K., Xiao X., Wu X., Liu L., She Y., Liu S., Zhou R., Yin G., Shao X., Dai Y., Lin H., Liu R. (2022). Peptide Polymer-Doped Cement Acting as an Effective Treatment of MRSA-Infected Chronic Osteomyelitis. Adv. Funct. Mater..

[cit22] Tavakoli M., Bakhtiari S. S. E., Karbasi S. (2020). Incorporation of chitosan/graphene oxide nanocomposite in to the PMMA bone cement: Physical, mechanical and biological evaluation. Int. J. Biol. Macromol..

[cit23] Singh J., Mandell J. B., Scheidt J., Orlando J. D., Kadir J. C., Ribnick W. B., Brothers K. M., Urish K. L., Sydlik S. A. (2025). Antibiotic TMA (Therapeutic Methacrylic) Comonomers: Synergizing Mechanical Integrity with Infection Control in Bone Cement. Adv. Funct. Mater..

[cit24] Wright Z. M., Pandit A. M., Holt B. D., Sydlik S. A. (2019). Therapeutic Methacrylic Comonomers for Covalently Controlled Release from Mechanically Robust Bone Cement: Kinetics and Structure–Function Relationships. Macromolecules.

[cit25] Wright Z. M., Holt B. D., Sydlik S. A. (2017). Covalently-controlled drug delivery *via* therapeutic methacrylic tissue adhesives. J. Mater. Chem. B.

[cit26] Xu Y., Lin H., Qi Y.-Y., Wang C., Han L.-X., He F., Sang H.-X., Chu J.-J. (2025). Point-of-care bone cement based on natural peptide comonomer protects orthopaedic implants from bacterial challenge. Mater. Today Bio.

[cit27] Xu Y., Lin H., Gao Z., Guo R., Kan Y.-C., Han L.-Y., Bu W.-H., Wang Z., Asilebieke A., Han L.-X., Li C., He F., Chu J.-J. (2024). Injectable isoniazid-loaded bone cement based on hydrazone bonds achieving long-term release and decent mechanical properties. J. Mater. Chem. B.

[cit28] Chu J., Li C., Guo J., Xu Y., Fu Y. (2022). Preparation of new bio-based antibacterial acrylic bone cement *via* modification with a biofunctional monomer of nitrofurfuryl methacrylate. Polym. Chem..

[cit29] Zhu W., Liu F., Yu B., He J. (2021). Preparation of antibacterial acrylic bone cement with methacrylate derived from benzothiazole. J. Mech. Behav. Biomed. Mater..

[cit30] Chen Y., Caneli G., Almousa R., Xie D. (2022). A novel antibacterial zirconia-containing PMMA bone cement. J. Mech. Behav. Biomed. Mater..

[cit31] Han L.-Y. , Yang D., Wang H.-L., Gao Z., Qi Y.-Y., Zhou T., Xu Y., and Chu J.-J., Bone cement prepared using the isobornyl acrylate monomer shows decent mechanical strength and antibacterial activity, New J. Chem., 2025, 49, 2106 2115, https://search.crossref.org/search/works?q=Bone+cement+prepared+using+the+isobornyl+acrylate+monomer+shows+decent+mechanical+strength+and+antibacterial+activity&from_ui=yes

[cit32] Bu W.-H., Asilebieke A., Han L.-Y., Xu Y., Zhou T., Chu J.-J. (2025). Preparation and characterization of novel PMMA bone cement containing 3,4-dichloro-5-hydroxyfuran-2(5H)-one. RSC Adv..

[cit33] Wang X., Bu W.-H., Han L.-Y., Han L.-X., Liang Q.-L., He S., Gao Z., Xu Y., Chu J.-J., He F. (2025). Furanone-based comonomer used to manufacture antibacterial bone cement with simultaneously enhanced mechanical strength and antibacterial activity. Mater. Adv..

[cit34] Zhu W., Liu F., He J. (2018). Effect of polymerizable quaternary ammonium monomer MEIM-x’s alkyl chain length and content on bone
cement's antibacterial activity and physicochemical properties. J. Mech. Behav. Biomed. Mater..

[cit35] Chen Y., Caneli G., Xie D. (2022). A PMMA bone cement with improved antibacterial function and flexural strength. J. Biomater. Sci., Polym. Ed..

[cit36] Abid C. K. V. Z., Jain S., Jackeray R., Chattopadhyay S., Singh H. (2017). Formulation and characterization of antimicrobial quaternary ammonium dendrimer in poly(methyl methacrylate) bone cement. J. Biomed. Mater. Res., Part B.

[cit37] Barbour A., Wescombe P., Smith L. (2020). Evolution of Lantibiotic Salivaricins: New Weapons to Fight Infectious Diseases. Trends Microbiol..

[cit38] Field D., Fernandez de Ullivarri M., Ross R. P., Hill C. (2023). After a century of nisin research—where are we now?. FEMS Microbiol. Rev..

[cit39] Bahrami A., Delshadi R., Jafari S. M., Williams L. (2019). Nanoencapsulated nisin: An engineered natural antimicrobial system for the food industry. Trends Food Sci. Technol..

[cit40] Wu J., Zang M., Wang S., Zhao B., Bai J., Xu C., Shi Y., Qiao X. (2023). Nisin: From a Structural and Meat Preservation Perspective. Food Microbiol..

[cit41] Yuan L. , Wu S., Tian K., Wang S., Wu H., and Qiao J., Nisin-Relevant Antimicrobial Peptides: Synthesis Strategies and Applications, Food Funct., 2024, 15, 9662 9677, https://search.crossref.org/search/works?q=Nisin-relevant+antimicrobial+peptides:+synthesis+strategies+and+applications&from_ui=yes 10.1039/d3fo05619h 39246095

[cit42] Dickman R., Danelius E., Mitchell S. A., Hansen D. F., Erdélyi M., Tabor A. B. A. (2019). Chemical Biology Approach to Understanding Molecular Recognition of Lipid II by Nisin (1–12): Synthesis and NMR Ensemble Analysis of Nisin (1–12) and Analogues. Chem. – Eur. J..

[cit43] Hsu S.-T. D., Breukink E., Tischenko E., Lutters M. A. G., de Kruijff B., Kaptein R., Bonvin A. M. J. J., van Nuland N. A. J. (2004). The Nisin–Lipid II Complex Reveals a Pyrophosphate Cage That Provides a Blueprint for Novel Antibiotics. Nat. Struct. Mol. Biol..

[cit44] Medeiros-Silva J., Jekhmane S., Paioni A. L., Gawarecka K., Baldus M., Swiezewska E., Breukink E., Weingarth M. (2018). High-Resolution NMR Studies of Antibiotics in Cellular Membranes. Nat. Commun..

[cit45] Paul C., Steinhauser E., Kühn K. D. (2023). Verarbeitungseigenschaften und Viskositäten von PMMA-Knochenzementen. Orthopadie.

