# Supplementary material for: One-step antimicrobial peptide immobilization technique: universal compatibility with clinical bone cements
Source: RSC Adv. 2026 Nov 12. Online ahead of print. doi: 10.1039/d6ra08377c (PMC13755755; doi:10.1039/d6ra08377c)
Supplement: Supplementary file 1 [file RA-OLF-D6RA08377C-s003.pdf]

Supplementary Information (SI) for RSC Advances.
This journal is © The Royal Society of Chemistry

Supplementary information

One-Step Antimicrobial Peptide Immobilization Technique: Universal Compatibility with Clinical Bone Cements

Supplementary Information contains:

Supplementary Tables S1–S2

Supplementary Figures S1–S7

Table S1. Bone cement formulations. (The total amount of bone cement is approximately 1g)

Formulation	Powder(mg)		Liquid(□L)
	PMMA	Drug	MMA
PALACOS® MV	638	0	385
PALACOS® MV-N	606	32	385
PALACOS® MV-GS	606	32	385
MT-HA	638	0	385
MT-HA-N	606	32	385
MT-HA-GS	606	32	385
J-MV-40	638	0	385
J-MV-40-N	606	32	385
J-MV-40-GS	606	32	385

Table S2. CPC Bone Cement Formulation. (The total amount of cement is approximately 1g)

Formulation	Powder(mg)		Liquid(□L)
	α-TCP	Nisin	2.5% Na_2HPO_4
CPC	700	0	300
CPC-N	668	32	300

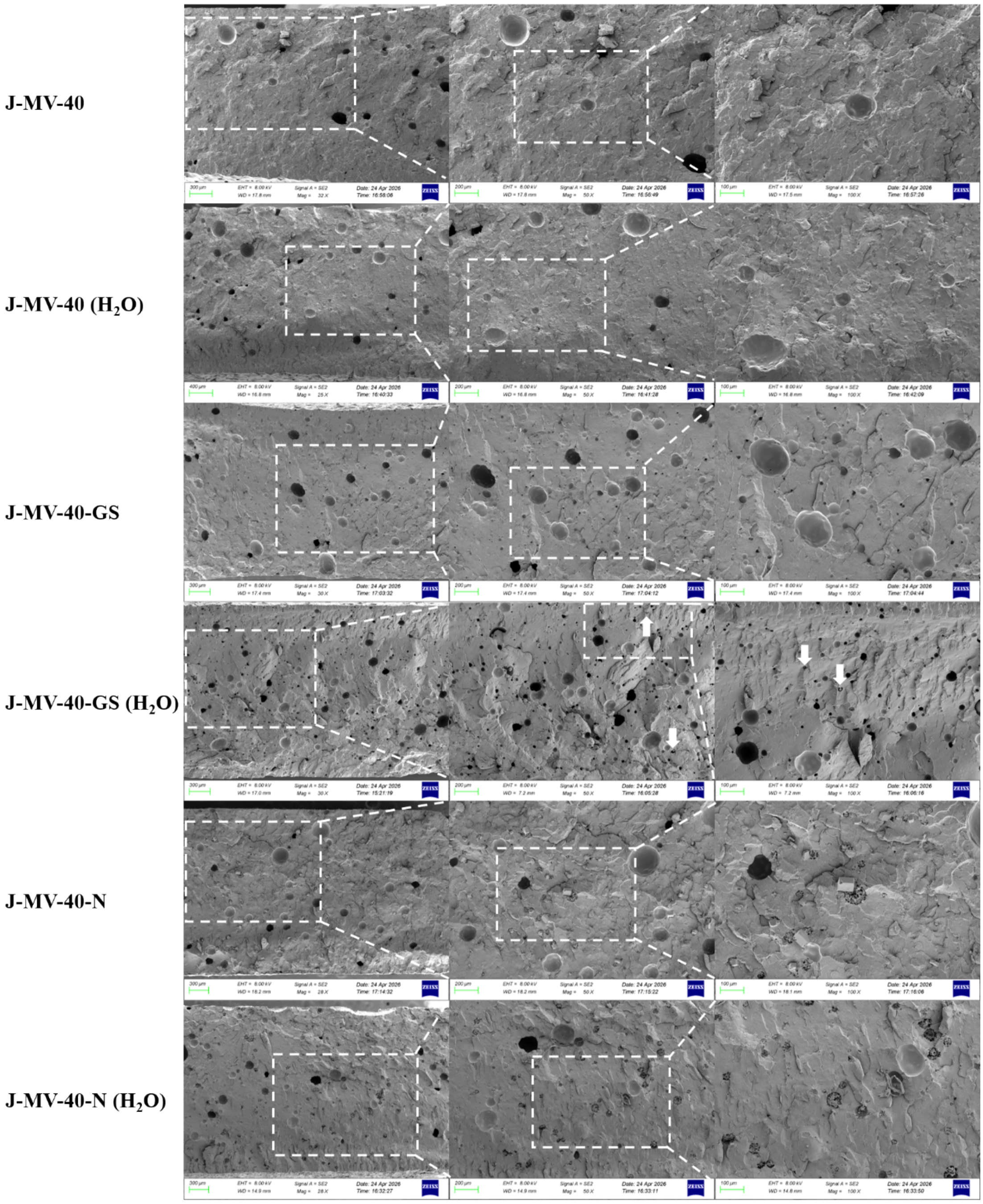

Figure S1. SEM images of the cross-sectional microstructures of PMMA, GS-loaded, and Nisin-loaded J-MV-40 bone cements before and after immersion. From left to right, the magnifications are 25–32×, 50×, and 100×, respectively. The suffix (H_2O) indicates the specimen after immersion in deionized water.

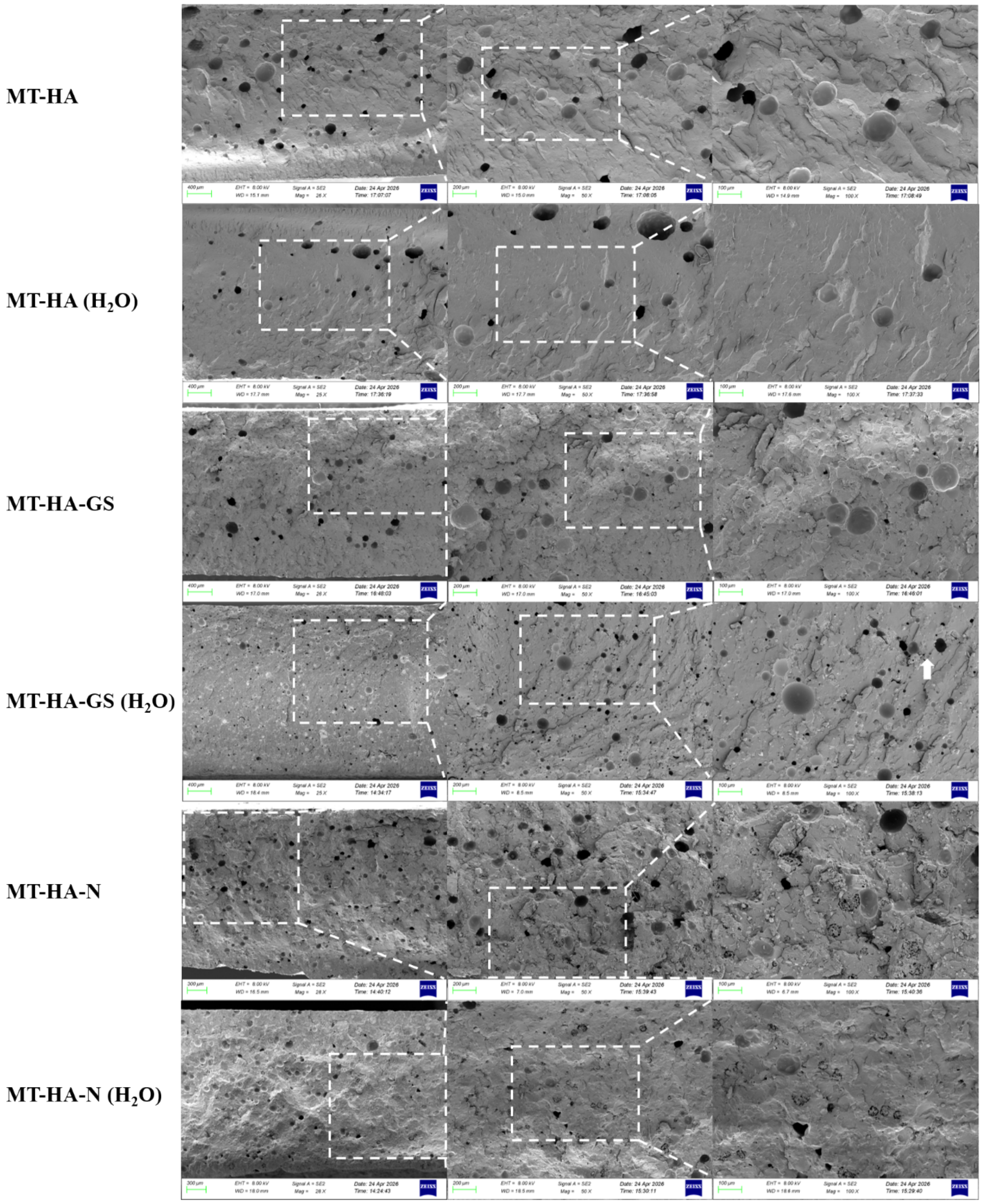

Figure S2. SEM images of the cross-sectional microstructures of PMMA, GS-loaded, and Nisin-loaded MT-HA bone cements before and after immersion. From left to right, the magnifications are 25–28×, 50×, and 100×, respectively. The 50× image of the MT-HA-GS (H_2O) cement is an enlarged view of the dashed-boxed region in the 25× image, rotated 180° clockwise. The 50× image of the MT-HA-N cement is an enlarged view of the dashed-boxed region in the 28× image, rotated 180° clockwise. The 50× image of the MT-HA-N (H_2O) cement is an enlarged view of the dashed-boxed region in the 28× image, rotated 180° clockwise. The suffix (H_2O) indicates the specimen after immersion in deionized water.

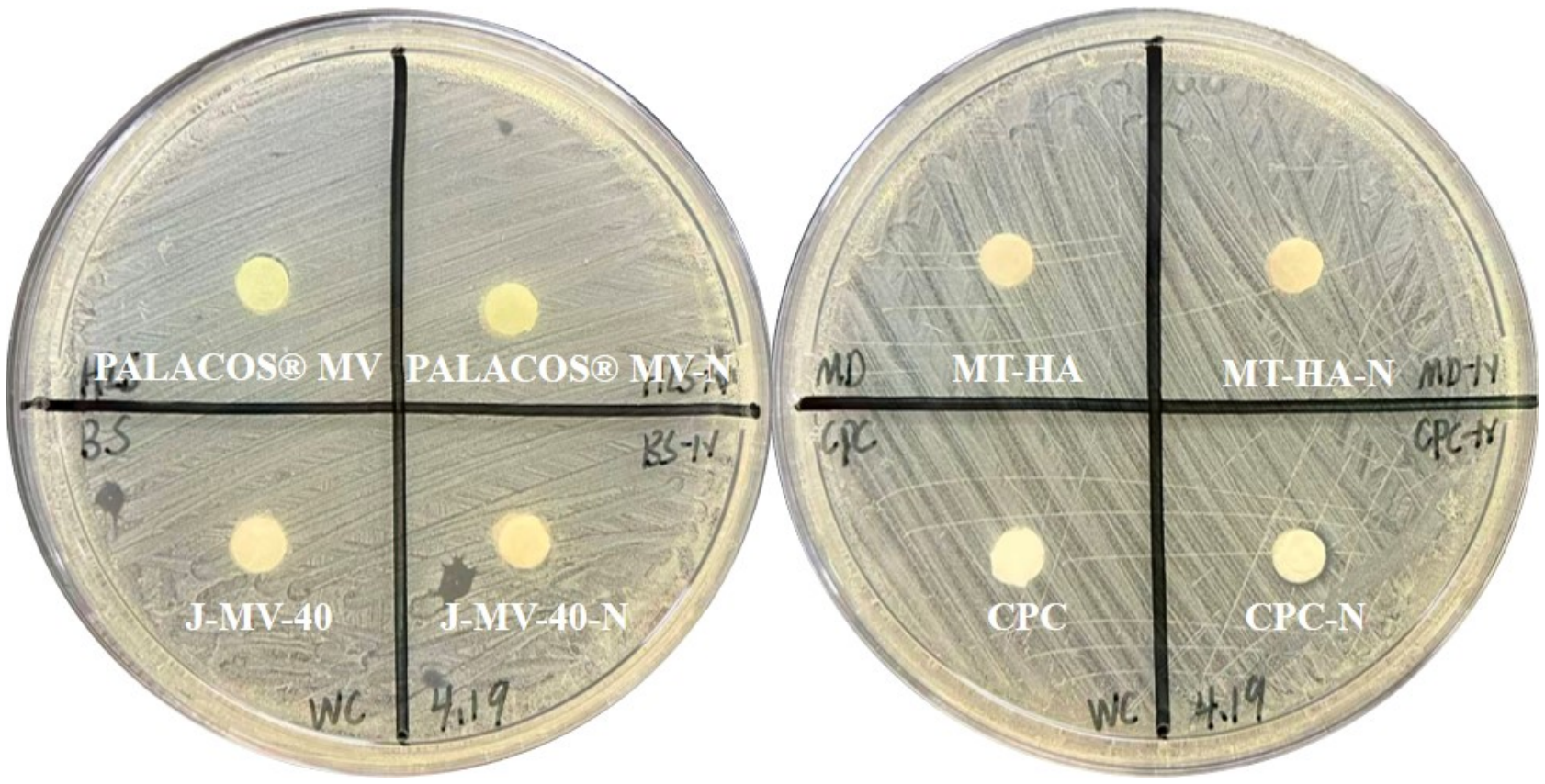

Figure S3. Inhibition zone of bone cements against *S. aureus.*

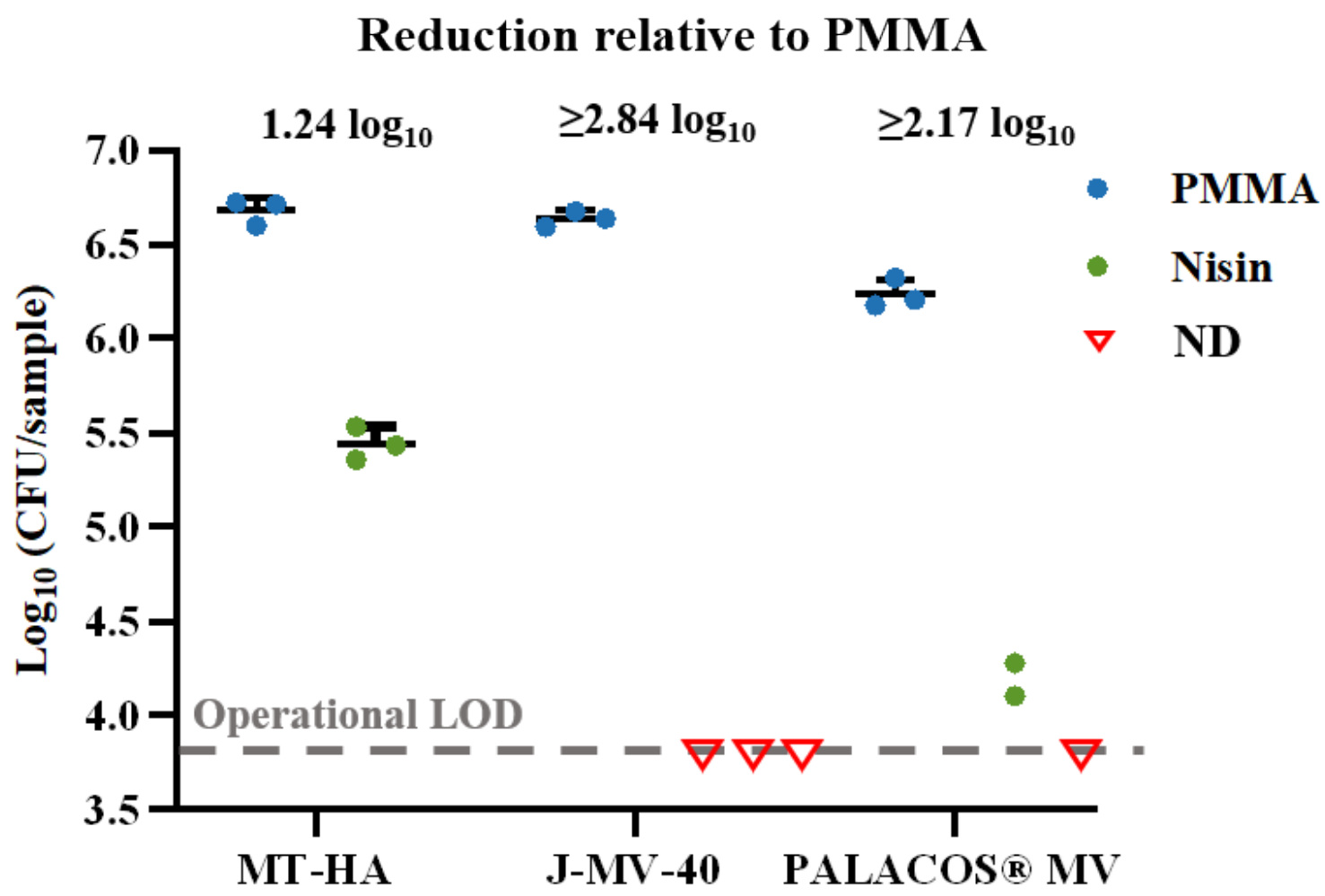

Fig. S4. Viable bacterial counts on three commercial cements before and after Nisin modification, expressed as log₁₀(CFU/sample). Each data point represents an independent sample (n = 3). ND indicates that no colonies were detected under the tested conditions. The dashed line represents the operational LOD. Log reductions were calculated relative to the corresponding unmodified bone cement controls, with the LOD used as a conservative substitute for non-detectable samples.

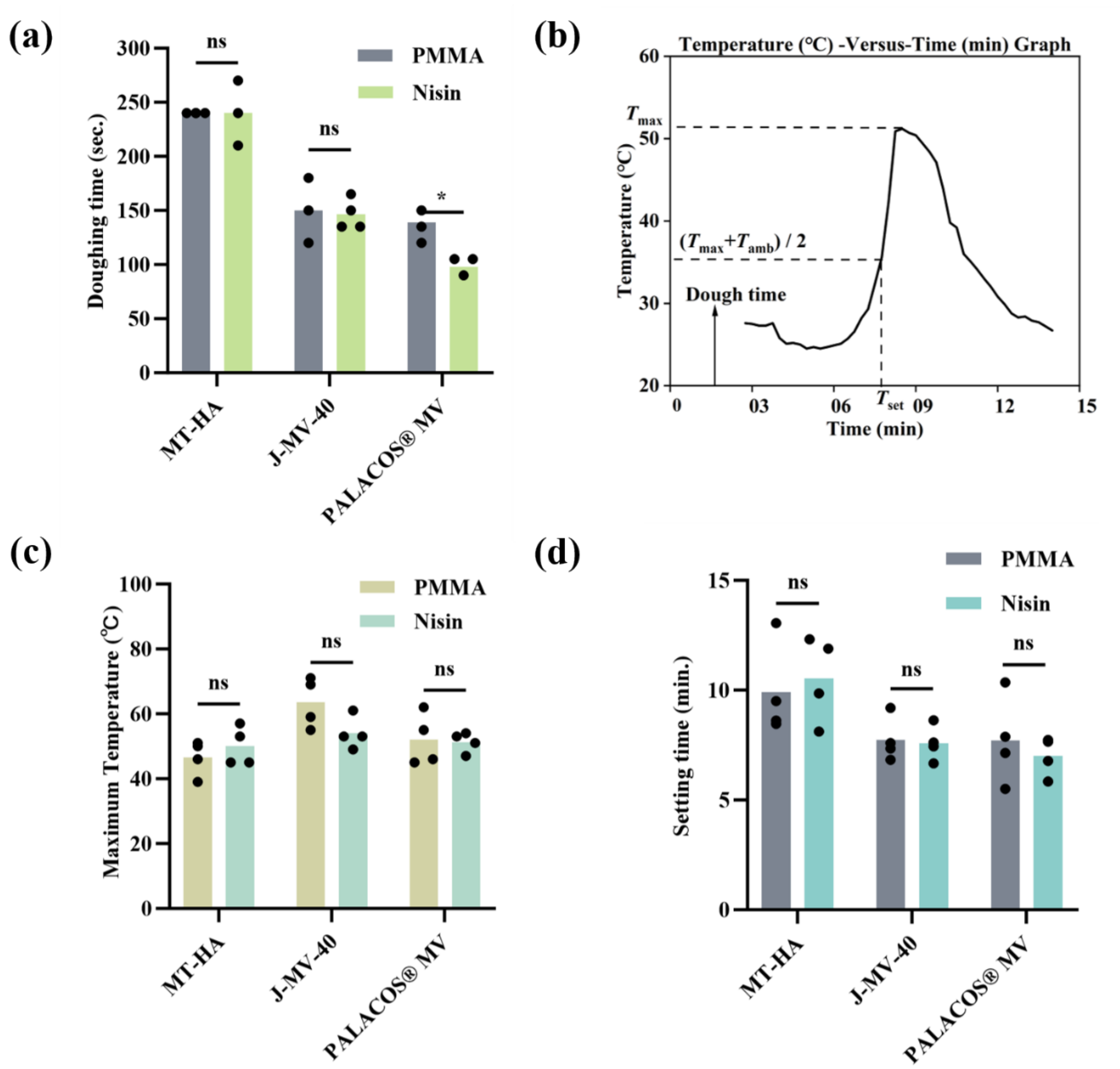

Figure S5. Handling performance using manufacturer-recommended powder-to-liquid ratios. (a) Doughing time. (b) Temperature–time curve. (c) Maximum temperature. (d) Setting time. Since the temperature–time curves of all groups exhibited similar variation trends, only one representative temperature–time curve of the PALACOS® MV-N cement is shown in the figure, while the complete temperature variation data for the remaining groups are provided in Supplementary File 2.

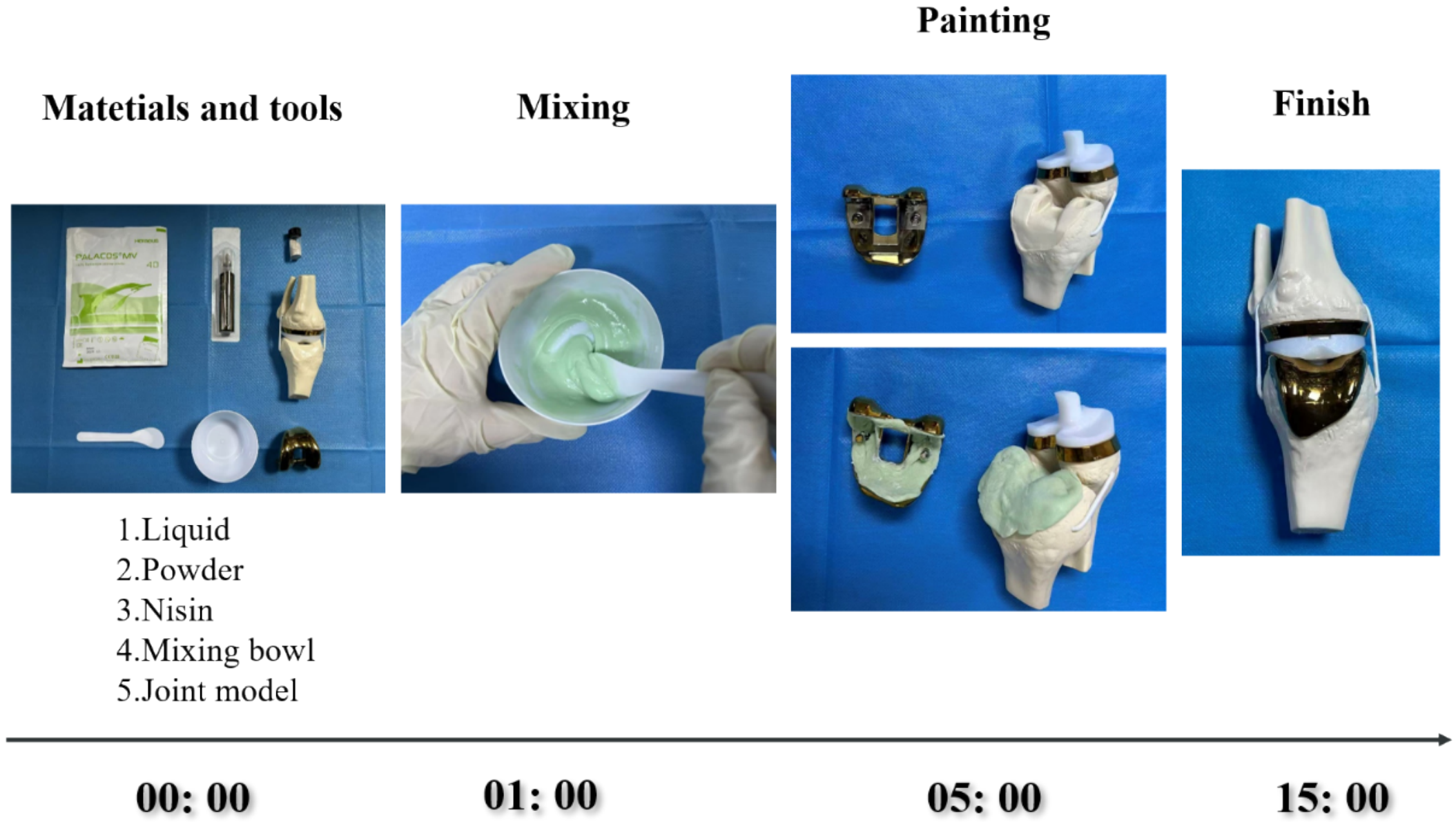

Figure S6. Operating room demonstration of PALACOS® MV-N bone cement.

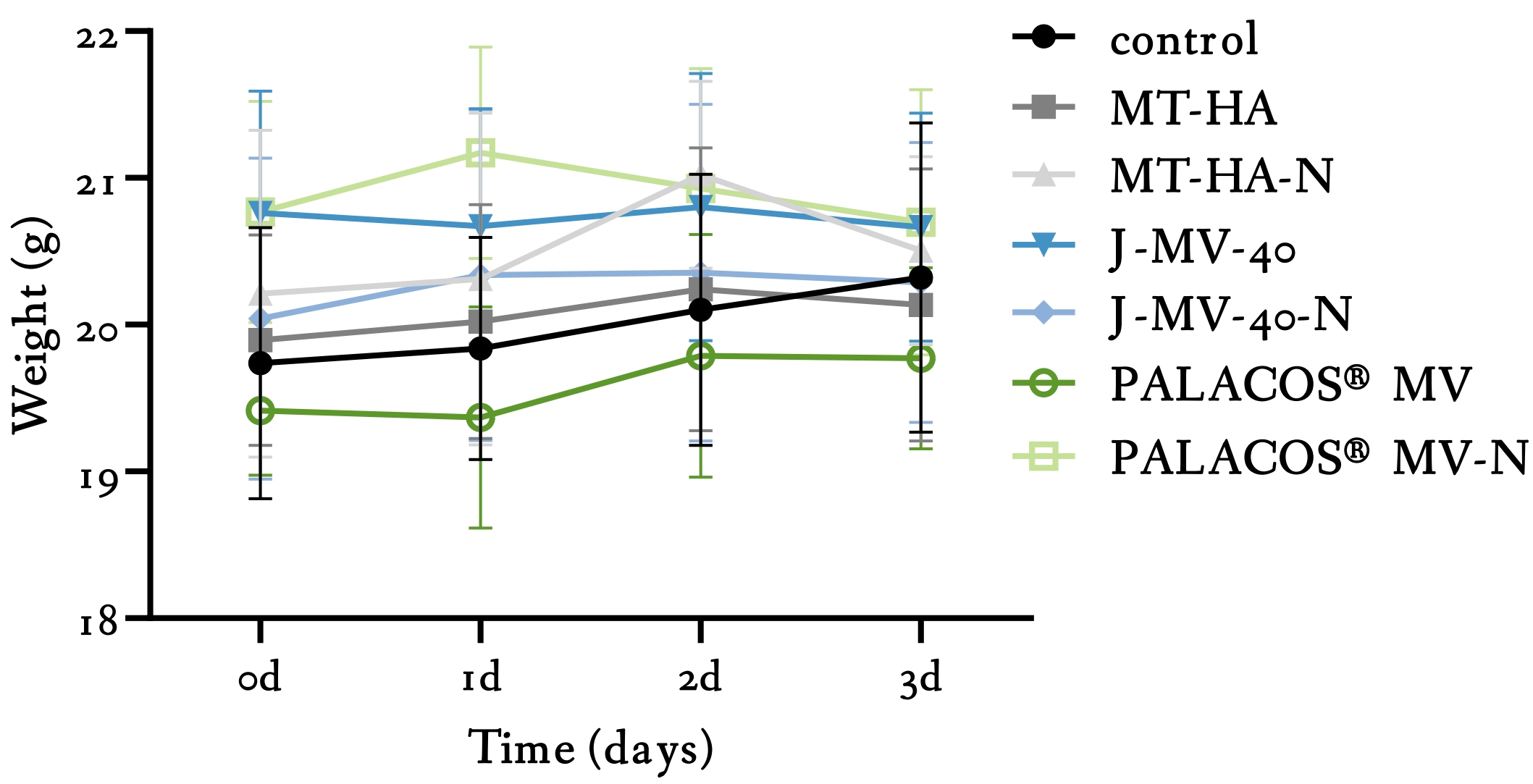

Figure S7. Body weight changes in mice over three consecutive days after intraperitoneal injection of bone cement extracts.
